# Supplementary material for: Comparative 4-year risk and type of hospital admission among homeless and housed emergency department attendees: longitudinal study of hospital records in England 2013–2018
Source: BMJ Open. 2021 Jul 26;11(7):e049811. doi: 10.1136/bmjopen-2021-049811 (PMC8314693; doi:10.1136/bmjopen-2021-049811)
Supplement: Supplementary data [file bmjopen-2021-049811supp001.pdf]

Supplementary Material

In HES A&E 2013/14, there are N = 18,517,381 observations on n = 11,936,228 patients

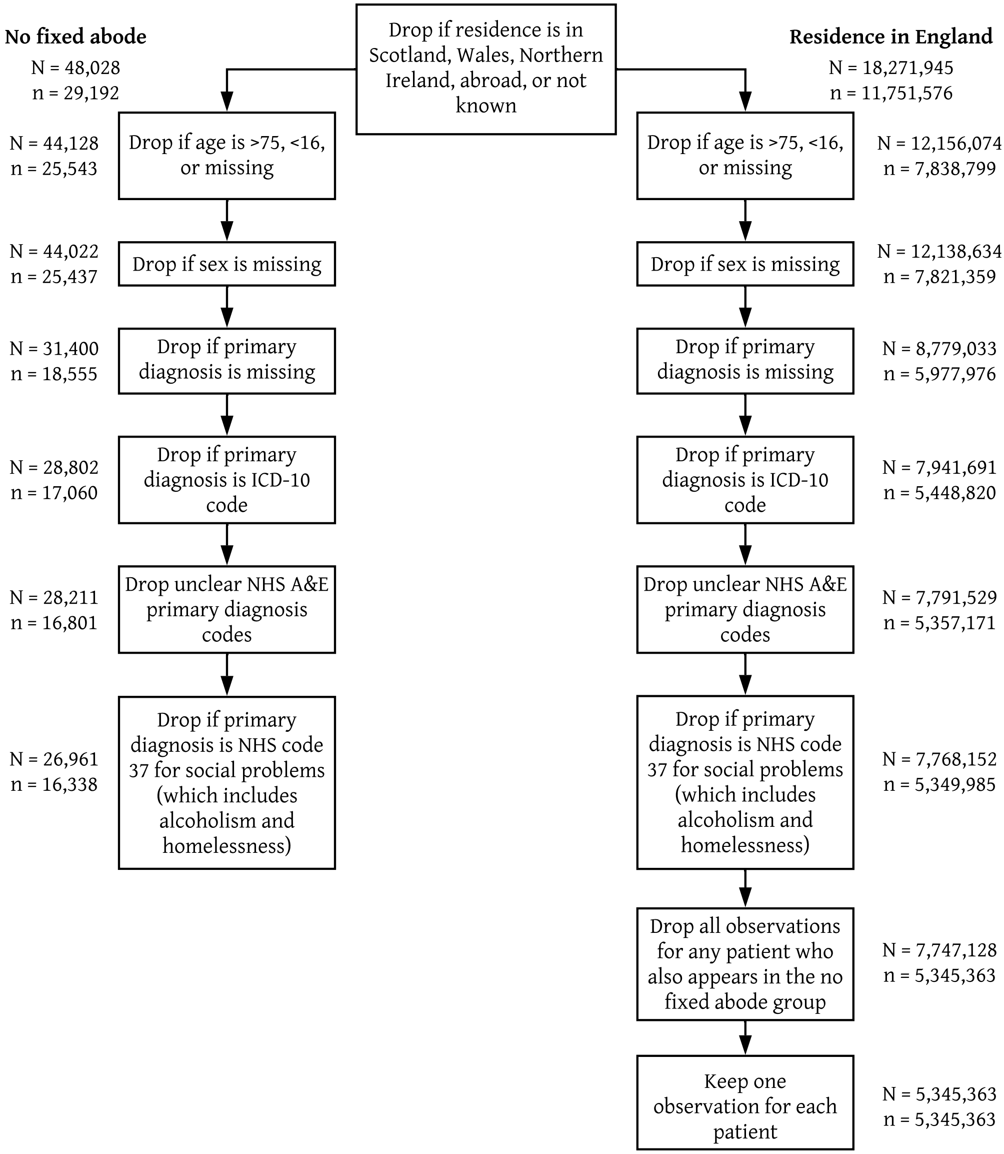

Pre-matching sample of 16,338 patients with no fixed abode and 5,345,363 housed patients
