# Supplementary material for: Comparative 4-year risk and type of hospital admission among homeless and housed emergency department attendees: longitudinal study of hospital records in England 2013–2018
Source: BMJ Open. 2021 Jul 26;11(7):e049811. doi: 10.1136/bmjopen-2021-049811 (PMC8314693; doi:10.1136/bmjopen-2021-049811)
Supplement: Author's manuscript [file bmjopen-2021-049811.draft_revisions.pdf]

BMJ Open is committed to open peer review. As part of this commitment we make the peer review history of every article we publish publicly available.

When an article is published we post the peer reviewers' comments and the authors' responses online. We also post the versions of the paper that were used during peer review. These are the versions that the peer review comments apply to.

The versions of the paper that follow are the versions that were submitted during the peer review process. They are not the versions of record or the final published versions. They should not be cited or distributed as the published version of this manuscript.

BMJ Open is an open access journal and the full, final, typeset and author-corrected version of record of the manuscript is available on our site with no access controls, subscription charges or pay-per-view fees (http://bmjopen.bmj.com).

If you have any questions on BMJ Open's open peer review process please email info.bmjopen@bmj.com

BMJ Open

Comparative four year risk and type of hospital admission among homeless and housed emergency department attendees: longitudinal study of hospital records in England 2013-2018

Journal:	*BMJ Open*
Manuscript ID	bmjopen-2021-049811
Article Type:	Original research
Date Submitted by the Author:	02-Feb-2021

Complete List of Authors:	Moss, Charlie; The University of Manchester, Health Organisation, Policy and Economics, Centre for Primary Care and Health Services Research, School of Health Sciences<br>Sutton, Matt; The University of Manchester, Health Organisation, Policy and Economics, Centre for Primary Care and Health Services Research, School of Health Sciences; The University of Melbourne, Melbourne Institute: Applied Economic and Social Research<br>Cheraghi-Sohi, Sudeh; The University of Manchester, NIHR Greater Manchester Patient Safety Translational Research Centre, Centre for Primary Care and Health Services Research, Manchester Academic Health Science Centre<br>Sanders, Caroline; The University of Manchester, NIHR Greater Manchester Patient Safety Translational Research Centre, Centre for Primary Care and Health Services Research, Manchester Academic Health Science Centre<br>Allen, Thomas; The University of Manchester, Manchester Centre for Health Economics, School of Health Sciences; University of Southern Denmark, Danish Centre for Health Economics, Department of Public Health

Keywords:	ACCIDENT & EMERGENCY MEDICINE, PUBLIC HEALTH, SOCIAL MEDICINE

SCHOLARONE™
Manuscripts

I, the Submitting Author has the right to grant and does grant on behalf of all authors of the Work (as defined in the below author licence), an exclusive licence and/or a non-exclusive licence for contributions from authors who are: i) UK Crown employees; ii) where BMJ has agreed a CC-BY licence shall apply, and/or iii) in accordance with the terms applicable for US Federal Government officers or employees acting as part of their official duties; on a worldwide, perpetual, irrevocable, royalty-free basis to BMJ Publishing Group Ltd ("BMJ") its licensees and where the relevant Journal is co-owned by BMJ to the co-owners of the Journal, to publish the Work in this journal and any other BMJ products and to exploit all rights, as set out in our licence.

The Submitting Author accepts and understands that any supply made under these terms is made by BMJ to the Submitting Author unless you are acting as an employee on behalf of your employer or a postgraduate student of an affiliated institution which is paying any applicable article publishing charge ("APC") for Open Access articles. Where the Submitting Author wishes to make the Work available on an Open Access basis (and intends to pay the relevant APC), the terms of reuse of such Open Access shall be governed by a Creative Commons licence – details of these licences and which Creative Commons licence will apply to this Work are set out in our licence referred to above.

Other than as permitted in any relevant BMJ Author's Self Archiving Policies, I confirm this Work has not been accepted for publication elsewhere, is not being considered for publication elsewhere and does not duplicate material already published. I confirm all authors consent to publication of this Work and authorise the granting of this licence.

For peer review only

Comparative four year risk and type of hospital admission among homeless and housed emergency department attendees: longitudinal study of hospital records in England 2013-2018

Charlie Moss,[1] Matt Sutton,[1,2] Sudeh Cheraghi-Sohi,[3] Caroline Sanders,[3] Thomas Allen[4,5]

[1] Health Organisation, Policy and Economics (HOPE), Centre for Primary Care and Health Services Research, School of Health Sciences, The University of Manchester, Manchester, UK

[2] Melbourne Institute: Applied Economic and Social Research, The University of Melbourne, Melbourne, Australia

[3] NIHR Greater Manchester Patient Safety Translational Research Centre, Centre for Primary Care and Health Services Research, The University of Manchester, Manchester Academic Health Science Centre, Manchester, UK

[4] Manchester Centre for Health Economics, School of Health Sciences, The University of Manchester, Manchester, UK

[5] Danish Centre for Health Economics, Department of Public Health, The University of Southern Denmark, Odense, Denmark

Corresponding author: Charlie Moss, Suite 9, 6th floor, Williamson Building, Oxford Road, The University of Manchester, Manchester, M13 9PL, UK

Email: charlie.moss@manchester.ac.uk

Keywords: Homelessness; Health service use; Emergency health care; Health care access

For peer review only

DECLARATIONS

Ethics approval and consent to participate

Not applicable

Consent for publication

Not applicable

Availability of data and materials

The Hospital Episode Statistics data that support the findings of this study are available from NHS Digital but restrictions apply to the availability of these data, which were used under license for the current study, and so are not publicly available.

Competing interests

The authors declare that they have no competing interests.

Funding

This research was funded jointly by a NIHR Research Methods Fellowship [RM-FI-2017-08-018] and the NIHR Greater Manchester Patient Safety Translational Research Centre [PSTRC-2016-003]. The views expressed are those of the authors and not necessarily those of the NIHR or the Department of Health and Social Care.

Patient and public involvement

Patients or the public were not involved in the design of this study.

For peer review only

ABSTRACT

Objectives: People experiencing homelessness are frequent users of secondary care. Currently, there is no study of potentially preventable admissions for homeless patients in England. We aim to estimate the number of potentially preventable hospital admissions for homeless patients and compare to housed patients with similar characteristics.

Design: Retrospective matched cohort study

Setting: Hospitals in England.

Participants: 16,338 homeless patients and 75,359 housed patients aged 16 to 75 who attended an emergency department in England in 2013/14, matched on the basis of age, sex, region, and primary diagnosis.

Primary and secondary outcome measures: Annual counts of admissions, emergency admissions, ambulatory care-sensitive (ACS) emergency admissions, acute ACS emergency admissions, and chronic ACS emergency admissions over the following four years (2014/15 to 2017/18). We additionally compare the prevalence of specific ACS conditions for homeless and housed patients.

Results: Mean admissions per 1000 patients per year were 470 for homeless patients and 230 for housed patients. Adjusted for confounders, annual admissions were 1.82 times higher (IRR=1.82; 95% CI [1.72 to 1.93]), emergency admissions 2.12 times higher (IRR=2.12; 95% CI [1.99 to 2.25]), and ACS admissions 1.70 times higher (IRR=1.70; 95% CI [1.56 to 1.85]), compared with housed patients. The effect was greater for acute (IRR=1.81; 95% CI [1.68 to 1.96]) than chronic (IRR=1.52; 95% CI [1.33 to 1.74]) ACS conditions. ACS conditions that were relatively more common for homeless patients were cellulitis, convulsions/epilepsy, diabetes, and chronic angina.

Conclusions: Homeless patients use hospital services at higher rates than housed patients, particularly emergency admissions. ACS admissions of homeless patients are higher which suggests some admissions may be potentially preventable with improved access to primary care. However, these admissions comprise a small share of total admissions.

For peer review only

Strengths and limitations of this study

- Large sample of 91,697 patients
- Comparative study design
- Use of linked mortality data to avoid immortality bias
- "No fixed abode" flag used to identify patients who are homeless is not a perfect indicator of homelessness
- Use of emergency department attendances data to identify study participants may affect generalisability to less-regular emergency department attendees

For peer review only

Background

Homelessness remains a problem in England, as in many developed countries.[1,2] The homelessness charity, Shelter, estimates that 307,000 people experienced homelessness in England in 2017,[3] although this is considered an underestimate as it does not count the vulnerably housed, or those missed by official statistics who often rely on friends and family.

Homelessness is associated with poor health and a high rate of mortality.[4–6] Homeless people often have comorbidities usually found in substantially older people.[1,7] Tri-morbidity,[8] is a term used to describe the common combination of physical ill-health, mental ill-health and drug or alcohol misuse which gives rise to complex needs. In 2018, the average age of death for homeless men and women in England and Wales was 45 and 43 years, respectively, compared with 76 and 81 years for the general population.[9] A 2019 study estimated that 30% of deaths amongst homeless people are from conditions amenable to health care, compared to 23% of deaths in a comparison group of housed people from the most deprived quintile of areas in England.[10]

There is some evidence that homeless people have limited access to routine health care. Homeless people face barriers to registering with and accessing mainstream primary care services,[11,12] often due to organisational barriers (e.g. inflexible appointment schedules) and previous negative experiences, including experience (or fear) of stigma when attempting to access services. People who are homeless are less likely to be registered with a GP than the general population.[13] It is possible that poor access to primary care contributes to the high use of emergency departments and inpatient care by people who are homeless,[7,14,15] which is both costly and lacking in continuity of care for patients who often have complex health problems. The latter is viewed to be especially important for meeting long-term needs and delivering care with compassion for homeless people who have common experiences of trauma.[16]

It has been proposed that emergency hospital admissions related to some conditions, known as ambulatory care-sensitive (ACS) conditions, could be avoided if timely and effective primary care was provided.[17,18] Diabetes, influenza, asthma, and anaemia are examples of such conditions. Elevated ACS hospital admissions may also provide indirect insights into the quality of primary care provided, although there are many aspects of quality of care that they will fail to capture.[19] One study has suggested that there were 1.77 million ACS admissions in England during 2011-2012, accounting for 10.9 million bed days.[20]

For peer review only

Currently, there is no study of potentially preventable admissions for homeless patients in England. We add to the evidence by using a comparative design: we use national data to match a cohort of homeless patients to a cohort of housed patients with similar observable characteristics. We compare the relative frequency of hospital admissions for each cohort in the following four years, and the prevalence of specific ACS conditions. We then discuss the extent to which these admissions may be preventable with improved access to primary care.

Methods

In England, administrative data are collected on National Health Service (NHS) hospital service use for all admissions, accident and emergency (A&E) attendances and outpatient appointments.[21] These Hospital Episode Statistics (HES) data contain information on diagnoses and treatment, as well as provider and patient characteristics. Each patient has a unique anonymised identifier which allows individual patients to be tracked when they are treated or seen in different hospital settings and over time.

Sample definition

We used HES A&E attendances data to identify two groups of patients: housed and homeless. Within HES there are fields derived from the patient's postcode which assign patients to different administrative geographies. We used the field for local authority district of residence, where those patients without a postcode are coded "no fixed abode".

We defined our sample of homeless patients as those who had an A&E attendance in the 2013/14 financial year and were recorded as having "no fixed abode" in the above field. The housed sample were those whose recorded local authority indicated that they resided in England.

For each A&E attendance in 2013/14, we retained the following variables required to match homeless and housed patients: sex, age, primary diagnosis and the location of the hospital attended.

We restricted our analysis to patients aged 16 to 75. We removed observations with missing or poor quality data in the fields used for matching. Where present, diagnosis codes are a

For peer review only

mixture of NHS A&E diagnosis condition codes[22] (92% amongst observations with "no fixed abode") and ICD-10 codes.[23]

We retained only the observations with NHS A&E diagnosis codes. We then coarsened the NHS codes down to their first two characters, corresponding to 39 broad diagnosis groups such as "head injury", "cardiac conditions", and "respiratory conditions".

Finally, we excluded those patients who had a primary diagnosis for "social problems" (NHS Accident and Emergency diagnosis code group 37; 3% of observations with "no fixed abode") in order to ensure that homeless patients were identified exclusively on the basis of their area of residence. This prevented homeless patients populating the 'housed' group via this diagnosis code.

We include a flowchart of observations removed at each stage in the supplementary material.

After the above exclusions, two mutually exclusive groups of patients remained: (1) 5,345,363 patients recorded as housed in England on all of their A&E attendances during 2013/14 and (2) 16,338 patients recorded as having no fixed abode on at least one of their A&E attendances during 2013/14.

Matching of homeless with housed patients

We matched each of the 16,338 homeless patients with up to five housed patients on combinations of sex, single year of age, primary diagnosis and clinical commissioning group (CCG) region of the emergency department the patient attended. Of the total 211 CCGs covering all of England, 109 were present. Many of the other 102 CCG areas did not contain a hospital with an A&E department. Others either had no attendances by patients with no fixed abode or observations were removed due to poor data quality.

After matching, the two cohorts comprised 16,338 homeless patients and 75,359 housed patients.

Outcomes

We used each matched patient's unique identifier to locate their admissions in the HES data for the following four financial years (2014/15, 2015/16, 2016/17, 2017/18).

We used two outcomes from the CCG Outcomes Indicator Set (CCG OIS)[24] to identify which emergency admissions were for an ACS condition: unplanned hospitalisation for chronic

For peer review only

ACS conditions (indicator 2.6) and emergency admission for acute conditions that should not usually require a hospital admission (indicator 3.1).

We constructed five counts for each patient in each financial year: admissions; emergency admissions; emergency admissions for any ACS condition; emergency admissions for an acute ACS condition; emergency admissions for a chronic ACS condition.

If a patient did not appear in the admissions data, we assigned a count of zero admissions for that financial year. Some patients died within the study period, and mortality is likely to differ between the housed and homeless cohorts. We therefore corrected this source of bias by using linked death records[25] to remove patients who die from the sample in the years following their death.

We estimated negative binomial regression models for each outcome, using pooled data for all four years. We present effects as Incident Rate Ratios (IRRs) and calculated marginal effects for the key variables to show the magnitude of these effects on the original scale. We used weights because each homeless patient is matched to between one and five housed patients. Each homeless patient has a weight of 1 and the weights assigned to housed patients are the reciprocal of the number of housed patients matched to the homeless patient.

For each outcome, we estimated a model which included a binary indicator for whether the patient has no fixed abode, year indicators, and indicators for the matching characteristics: sex, region of A&E attended in 2013/14, primary diagnosis group at A&E attendance in 2013/14, and ten-year age categories to allow for an expected non-linear relationship between age and all outcomes. We used robust standard errors clustered at the individual level.

For peer review only

Results

Matching of homeless with housed patients

We were able to match all 16,338 homeless patients to at least one housed patient. Prior to matching, the homeless group were younger, more likely to be male and were more likely to attend A&E in a London hospital (Table 1). Whilst attendances were distributed relatively similarly across the two populations in terms of primary diagnoses, there were a few notable exceptions. First, poisonings (which include overdoses) comprised a higher share of

attendances in homeless patients compared with housed: a homeless patient's attendance was for a diagnosis of poisoning 14 times more often. Second, homeless patient's attendances were for psychiatric conditions 9 times more often.

Conversely, acute injuries (such as laceration, sprain, dislocation and contusion) were typically more likely for housed patients compared with homeless patients. After matching, the two groups were markedly more balanced in terms of their characteristics.

Outcomes

The mean values of all outcomes were greater for homeless patients than for housed patients, both before and after matching (Table 2).

Each year, most people in the homeless and housed cohorts did not have any admissions. However, for all outcomes, patients in the homeless cohort more frequently had at least one admission each year, in comparison with the housed patients, both before and after matching.

A markedly greater percentage of the homeless cohort had a very large number of admissions and emergency admissions per year, compared with the housed cohort; 0.74% of the homeless cohort had ten or more admissions each year, compared with 0.12% of the matched housed cohort, and 0.69% of the homeless cohort had ten or more emergency admissions each year, compared with 0.09% of the housed cohort.

Table 3 presents the estimates from the negative binomial regression models. When the covariates are matched across groups, people with no fixed abode have a higher count of all outcomes (Table 3; Figure 1). For homeless patients, annual admissions were 1.82 times higher (IRR=1.82; 95% confidence interval (CI) [1.72 to 1.93]), emergency admissions 2.12 times higher (IRR=2.12; 95% CI [1.99 to 2.25]), and ACS admissions 1.70 times higher (IRR=1.70; 95% CI [1.56 to 1.85]), compared with housed patients. The effect was greater for acute (IRR=1.81; 95% CI [1.68 to 1.96]) than chronic (IRR=1.52; 95% CI [1.33 to 1.74]) ACS conditions.

The largest marginal effect of having no fixed abode was on emergency admissions; the homeless cohort had 0.231 additional emergency admissions (95% CI 0.204 to 0.259) and 0.027 additional ACS admissions (95% CI 0.021 to 0.032) each year compared with the housed cohort.

For peer review only

Admissions for several specific ACS conditions were relatively more common for the homeless cohort than for the housed cohort (Table 4). Of the acute ACS conditions, the percentage of total homeless ACS admissions that were for cellulitis was 1.6 times that of the housed cohort (18% compared with 11%), and the percentage for convulsions and epilepsy was 2.4 times that of the housed cohort (12% compared with 5%). Of the chronic ACS conditions, the percentage of homeless ACS admissions for diabetes was 1.3 times that of the housed cohort (9% compared with 7%) and the percentage for angina was twice that of the housed cohort (8% compared with 4%).

Discussion

Summary of main findings

Homeless patients had more admissions in general, more emergency admissions and more admissions for an ACS condition than housed patients with similar characteristics. This effect was greater for acute ACS conditions than for chronic ACS conditions.

We estimated that, compared to 1,000 housed patients with similar characteristics, 1,000 homeless patients would collectively have 231 more emergency admissions per year, and 27 more ACS emergency admissions per year. This suggests that ACS admissions account for around 12% of the additional emergency admissions experienced by homeless patients.

Acute ACS conditions for which emergency admission was more common for the homeless cohort were cellulitis and convulsions/epilepsy, and chronic ACS conditions were diabetes and angina.

Explanation of notable findings

Emergency admission for chronic angina was twice as common in the homeless cohort compared to the housed cohort. This could be due to the relatively high prevalence of cardio-vascular disease (CVD) in the homeless population,[6] and/or poor subsequent management of CVD in homeless patients compared with housed patients. The relatively high number of emergency admissions for diabetes in the homeless cohort suggests that, although the prevalence of diabetes is similar in homeless and housed patients,[26] it may be more poorly managed in homeless patients.

For peer review only

The higher number of acute ACS admissions for homeless patients is driven partly by emergency admissions for cellulitis in particular, and the causes of this are likely to be complex. Hospital admissions related to cellulitis are common amongst individuals who inject illicit drugs,[27,28] and homelessness is more common amongst individuals with problematic substance misuse.[29] The differential prevalence of cellulitis in the homeless cohort may therefore be indicative of increased rates of drugs misuse. This may also be tentatively indicated by the higher prevalence of admissions for convulsions, and the higher prevalence of A&E attendances for poisonings in the homeless cohort. Cellulitis is also strongly associated with comorbidity and conditions such as diabetes, however the causal relationship is likely to be bi-directional.[27] Furthermore, cellulitis is difficult to diagnose in primary care and even if patients attend primary care, these difficulties may lead to delays in treatment and subsequent and appropriate use of emergency healthcare.[30]

There is likely to be a complex interplay of factors at multiple levels which lead to the differences observed. People experiencing homelessness face barriers to accessing healthcare at the macro-level, such as potential disincentives for providers to provide more accessible services.[15] At the meso-level, organisational factors such as where, how and when appointments are offered in primary care place further barriers to care.[11,12] Finally, at the micro-level, both patient and professional factors are relevant. People experiencing homelessness have been described as having chaotic lifestyles.[15] In combination with the perceived and/or experienced stigma and discrimination from health professionals, this may lead to poor or delayed engagement with the health service. These social and structural issues mean that health problems can degenerate into more serious problems that subsequently require contact with emergency services.

Comparison with existing research

A study of homeless patients admitted to hospital in California in 2010 found that the odds that the admission was ACS was increased when the patient was black, admitted from the emergency department or transferred from another health care facility, or did not have Medicare.[31] A study of hospital inpatient records in Ireland from 2005-2014 found that of 2,051 emergency admissions for patients with “no fixed abode”, 280 (13.7%) were for an ACS condition. The most common ACS conditions amongst the admissions were convulsions/epilepsy (32.9%), cellulitis (22.1%) and COPD (10.4%).[32] Neither study compared ACS admissions of homeless patients with those of housed patients.

For peer review only

We add to the current evidence by using a comparative design: we matched a group of homeless patients to a group of housed patients with similar observable characteristics. We also used more recent data so our analysis is reflective of factors which have changed in the 2010s such as economic policies enacted by the UK government and the increase in homelessness in England.[33]

Limitations

The no fixed abode flag in HES is not a perfect indicator of homelessness. Some patients are recorded as having no fixed abode on one or more attendances and as being homed on other attendances. In part this will be the result of genuine changes in housing status, but we also suspect that false information may be provided in some cases, such as the address of a friend or relative.

Several factors may affect the generalisability of our study. First, it is possible that patients recorded as having no fixed abode are some of the most vulnerable and isolated homeless people who do not have an address to give. Second, because we selected patients to include in the study on the basis of A&E attendances, these patients (both homeless and housed) may be more likely to be admitted in an emergency than less regular A&E attendees.

We matched homeless patients to housed patients on the basis of sex, age, geographic location, and diagnosis received at their A&E attendance in 2013/14. It is highly likely that there are other unobserved differences between the two cohorts, other than homelessness. However, matching with this level of precision allowed us to match all 16,338 homeless patients to at least one housed patient, and provided a cohort of housed patients with sufficient similarity to the homeless cohort to enable a meaningful comparison.

Implications for policy and research

Dixon-Woods et al. have previously drawn attention to the complex interplay of factors (perceptions of need, priorities, attitudes of staff, resources, policies) that create inequalities and vulnerabilities for marginalised and disadvantaged groups in terms of access to healthcare.[35] Additional research has highlighted this complex interplay of barriers regarding access to primary care that can be viewed as creating a two-tier system for marginalised or 'hard-to-reach' groups.[36,37]

For peer review only

Our results suggest that patients with no fixed abode are admitted to hospital more frequently than housed patients for conditions classified as suitable for treatment and management in a primary care setting. Addressing structural factors such as improved access to primary care could potentially reduce some of these admissions. However, the fact that only 12% of the additional emergency admissions experienced by the homeless cohort were ACS suggests that other approaches are needed if the goal is to reduce homeless persons' admissions to hospital. For example, Reilly et al. have suggested that out-of-hours mobile medical units could be explored as a way of reducing A&E attendances by people who are homeless. [38]

Additionally, the likely relevance of other micro-level factors as a driver of some of the ACS admissions is a reminder of the complexity of health problems experienced by people who are homeless, which often have causes that are outside of the control of the healthcare system. The link between ACS admissions and access to primary care is less clear for this population than it might be for others.

Authors' contributions

SCS and CS conceived the original idea. MS and TA designed the empirical approach. CM carried out data analysis and drafted the first version of the manuscript. All authors contributed heavily to interpretation of results and editing the manuscript for the final version.

References

1. Fazel, S., Geddes, J. R. & Kushel, M. The health of homeless people in high-income countries: descriptive epidemiology, health consequences, and clinical and policy recommendations. *The Lancet* **384**, 1529–1540 (2014).
2. Lancet, T. A shared future for all: let's talk about homelessness. *The Lancet* **391**, 179 (2018).
3. Shelter. *Far from alone: homelessness in Britain in 2017*. (2017).

For peer review only

4. Morrison, D. S. Homelessness as an independent risk factor for mortality: results from a retrospective cohort study. *Int. J. Epidemiol.* **38**, 877–883 (2009).
5. Aldridge, R. W. *et al.* Morbidity and mortality in homeless individuals, prisoners, sex workers, and individuals with substance use disorders in high-income countries: a systematic review and meta-analysis. *The Lancet* **391**, 241–250 (2018).
6. Nanjo, A. *et al.* Prevalence, incidence, and outcomes across cardiovascular diseases in homeless individuals using national linked electronic health records. *Eur. Heart J.* **41**, 4011–4020 (2020).
7. Bowen, M. *et al.* Multimorbidity and emergency department visits by a homeless population: a database study in specialist general practice. *Br. J. Gen. Pract.* **69**, e515–e525 (2019).
8. Pathway. Homeless and inclusion health standards for commissioners and service providers. https://www.pathway.org.uk/wp-content/uploads/Version-3.1-Standards-2018-Final.pdf (2018).
9. Office for National Statistics. Deaths of homeless people in England and Wales: 2018. https://www.ons.gov.uk/peoplepopulationandcommunity/birthsdeathsandmarriages/deaths/bulletins/deathsofhomelesspeopleinenglandandwales/2018 (2019).
10. Aldridge, R. W. *et al.* Causes of death among homeless people: a population-based cross-sectional study of linked hospitalisation and mortality data in England. *Wellcome Open Res.* **4**, (2019).
11. Rae, B. E. & Rees, S. The perceptions of homeless people regarding their healthcare needs and experiences of receiving health care. *J. Adv. Nurs.* **71**, 2096–2107 (2015).
12. Gunner, E. *et al.* Provision and accessibility of primary healthcare services for people who are homeless: a qualitative study of patient perspectives in the UK. *Br. J. Gen. Pract.* **69**, e526–e536 (2019).

For peer review only

13. Elwell-Sutton, T., Fok, J., Albanese, F., Mathie, H. & Holland, R. Factors associated with access to care and healthcare utilization in the homeless population of England. *J. Public Health* **39**, 26–33 (2017).

14. Hewett, N. *Closing the gap through changing relationships*. https://www.health.org.uk/sites/default/files/PromotingCompassionateHealthcareHomelessPeople_report.pdf (2013).

15. Office of the Chief Analyst. *Healthcare for single homeless people*. https://www.housinglin.org.uk/_assets/Resources/Housing/Support_materials/Other_reports_and_guidance/Healthcare_for_single_homeless_people.pdf (2010).

16. Keats, H., Maguire, N., Johnson, R. & Cockersell, P. Psychologically informed services for homeless people, good practice guide. (2012).

17. Billings, J. *et al.* Impact Of Socioeconomic Status On Hospital Use In New York City. *Health Aff. (Millwood)* **12**, 162–173 (1993).

18. Sanderson, C. & Dixon, J. Conditions for Which Onset or Hospital Admission is Potentially Preventable by Timely and Effective Ambulatory Care. *J. Health Serv. Res. Policy* **5**, 222–230 (2000).

19. Hodgson, K., Deeny, S. R. & Steventon, A. Ambulatory care-sensitive conditions: their potential uses and limitations. *BMJ Qual. Saf.* **28**, 429–433 (2019).

20. Busby, J., Purdy, S. & Hollingworth, W. How do population, general practice and hospital factors influence ambulatory care sensitive admissions: a cross sectional study. *BMC Fam. Pract.* **18**, 67 (2017).

21. NHS Digital. Hospital Episode Statistics (HES). https://digital.nhs.uk/data-and-information/data-tools-and-services/data-services/hospital-episode-statistics (2019).

For peer review only

22. NHS. Accident and emergency diagnosis tables. *Data Dictionary* https://www.datadictionary.nhs.uk/web_site_content/supporting_information/clinical_coding/accident_and_emergency_diagnosis_tables.asp?shownav=1 (2020).

23. World Health Organisation. International classification of diseases and related health problems: 10th revision. (2016).

24. NHS Digital. Clinical Commissioning Group Outcomes Indicator Set (CCG OIS). *NHS Digital* https://digital.nhs.uk/data-and-information/publications/statistical/ccg-outcomes-indicator-set.

25. Office for National Statistics. Deaths. https://www.ons.gov.uk/peoplepopulationandcommunity/birthsdeathsandmarriages/deaths#datasets.

26. Lewer, D. *et al.* Health-related quality of life and prevalence of six chronic diseases in homeless and housed people: a cross-sectional study in London and Birmingham, England. *BMJ Open* **9**, e025192 (2019).

27. Wright, T. *et al.* Prevalence and severity of abscesses and cellulitis, and their associations with other health outcomes, in a community-based study of people who inject drugs in London, UK. *PLOS ONE* **15**, e0235350 (2020).

28. Marks, M. *et al.* Needles and the damage done: Reasons for admission and financial costs associated with injecting drug use in a Central London Teaching Hospital. *J. Infect.* **66**, 95–102 (2013).

29. Bramley, G. *et al. Hard edges: mapping severe and multiple disadvantage in England.* (2015).

30. Patel, M. *et al.* An interview study to determine the experiences of cellulitis diagnosis amongst health care professionals in the UK. *BMJ Open* **10**, e034692 (2020).

For peer review only

31. White, B. M., Ellis Jr, C. & Simpson, K. N. Preventable hospital admissions among the homeless in California: A retrospective analysis of care for ambulatory care sensitive conditions. *BMC Health Serv. Res.* **14**, 511 (2014).

32. O'Farrell, A., Evans, D. & Allen, M. The epidemiology of emergency in-patient hospitalisations among those with 'no fixed abode' (homeless) 2005-2014: what lessons can be learnt. *Ir. Med. J.* **109**, (2016).

33. Office for National Statistics. *UK homelessness: 2005 to 2018*. https://www.ons.gov.uk/peoplepopulationandcommunity/housing/articles/ukhomelessness/2005to2018 (2019).

34. Mcintyre, D., Thiede, M. & Birch, S. Access as a policy-relevant concept in low- and middle-income countries. *Health Econ. Policy Law* **4**, 179–193 (2009).

35. Dixon-Woods, M. *et al.* Conducting a critical interpretive synthesis of the literature on access to healthcare by vulnerable groups. *BMC Med. Res. Methodol.* **6**, 35 (2006).

36. Bristow, K. *et al.* Help Seeking and Access to Primary Care for People from "Hard-to-Reach" Groups with Common Mental Health Problems. *International Journal of Family Medicine* https://www.hindawi.com/journals/ijfm/2011/490634/ (2011) doi:https://doi.org/10.1155/2011/490634.

37. O'Donnell, P., Tierney, E., O'Carroll, A., Nurse, D. & MacFarlane, A. Exploring levers and barriers to accessing primary care for marginalised groups and identifying their priorities for primary care provision: a participatory learning and action research study. *Int. J. Equity Health* **15**, (2016).

38. Reilly, J., Hassanally, K., Budd, J. & Mercer, S. Accident and emergency department attendance rates of people experiencing homelessness by GP registration: a retrospective analysis. *BJGP Open* (2020) doi:10.3399/bjgpopen20X101089.

For peer review only

Table 1: Characteristics of housed and no fixed abode groups before and after matching

	Before matching			After matching		
	No fixed abode mean	Homed mean	Difference	No fixed abode mean	Homed mean	Difference
Individual characteristics						
Female (%)	24 (43)	49 (50)	-25	24 (43)	24 (43)	0
Age (years)	38.02 (13.66)	41.45 (16.84)	-3.43	38.02 (13.66)	37.67 (13.66)	0.35
Government office region of attendance (%)						
East	13 (33)	9 (28)	4	13 (33)	13 (33)	0
East Midlands	3 (17)	8 (27)	-5	3 (17)	3 (17)	0
London	31 (46)	16 (37)	15	31 (46)	30 (46)	1
North East	1 (10)	4 (20)	-3	1 (10)	1 (10)	0
North West	21 (41)	19 (39)	2	21 (41)	22 (41)	-1
South East	16 (36)	13 (34)	3	16 (36)	16 (37)	0
South West	5 (21)	9 (29)	-4	5 (21)	5 (21)	0
West Midlands	5 (23)	9 (28)	-4	5 (23)	5 (23)	0
Yorkshire & the Humber	5 (23)	12 (33)	-7	5 (23)	6 (23)	-1
Primary diagnosis group code (%) [a]						
Laceration (01)	5 (22)	6 (24)	-1	5 (22)	5 (23)	0
Contusion/abrasion (02)	3 (17)	4 (20)	-1	3 (17)	3 (17)	0
Soft tissue inflammation (03)	2 (15)	6 (24)	-4	2 (15)	3 (16)	-1
Head injury (04)	4 (21)	3 (17)	1	4 (21)	5 (21)	-1
Dislocation/fracture/joint injury/amputation (05)	4 (19)	7 (26)	-3	4 (19)	4 (20)	0
Sprain/ligament injury (06)	3 (16)	7 (26)	-4	3 (16)	3 (17)	0
Muscle/tendon injury (07)	2 (13)	3 (17)	-1	2 (13)	2 (13)	0
Nerve injury (08)	0.1 (3)	0.2 (5)	-0.1	0.1 (3)	0.1 (2)	0
Vascular injury (09)	0.1 (3)	0.1 (3)	0	0.1 (3)	0.02 (2)	0.08
Burns and scalds (10)	0.4 (6)	1 (9)	-0.6	0.4 (6)	0.3 (6)	0.1
Electric shock (11)	0.02 (2)	0.1 (3)	-0.08	0.02 (2)	0.03 (2)	-0.01
Foreign body (12)	0.5 (7)	1 (12)	-0.5	0.5 (7)	0.5 (7)	0

For peer review only

Bites/stings (13)	0.3 (6)	1 (10)	-0.7	0.3 (6)	0.3 (5)	0
Poisoning (inc. overdose) (14)	14 (35)	1 (12)	13	14 (35)	12 (32)	2
Near drowning (15)	0.01 (1)	0.01 (1)	0	0.01 (1)	0.003 (1)	0.007
Visceral injury (16)	0.1 (2)	0.05 (2)	0.05	0.1 (2)	0.03 (2)	0.07
Infectious disease (17)	1 (9)	1 (9)	0	1 (9)	1 (9)	0
Local infection (18)	2 (14)	2 (15)	0	2 (14)	2 (14)	0
Septicaemia (19)	0.1 (3)	0.2 (4)	-0.1	0.1 (3)	0.1 (2)	0
Cardiac conditions (20)	2 (15)	4 (19)	-2	2 (15)	3 (16)	-1
Cerebro-vascular conditions (21)	0.3 (6)	1 (9)	-0.7	0.3 (6)	0.3 (5)	0
Other vascular conditions (22)	0.5 (7)	1 (8)	-0.5	0.5 (7)	0.3 (5)	0.2
Haematological conditions (23)	0.2 (4)	0.3 (6)	-0.1	0.2 (4)	0.1 (3)	0.1
Central nervous system conditions (exc. Strokes) (24)	3 (17)	2 (14)	1	3 (17)	3 (17)	0
Respiratory conditions (25)	2 (14)	3 (17)	-1	2 (14)	2 (15)	0
Gastrointestinal conditions (26)	4 (20)	6 (23)	-2	4 (20)	5 (21)	-1
Urological conditions (inc. cystitis) (27)	1 (12)	2 (16)	-1	1 (12)	1 (12)	0
Obstetric conditions (28)	0.2 (4)	1 (7)	-0.8	0.2 (4)	0.2 (4)	0
Gynaecological conditions (29)	0.5 (7)	2 (13)	-1.5	0.5 (7)	0.5 (7)	0
Diabetes and other endocrinological conditions (30)	0.3 (6)	0.4 (6)	-0.1	0.3 (6)	0.2 (4)	0.1
Dermatological conditions (31)	0.5 (7)	1 (9)	-0.5	0.5 (7)	0.4 (6)	0.1
Allergy (inc. anaphylaxis) (32)	0.2 (5)	1 (8)	-0.8	0.2 (5)	0.1 (4)	0.1
Facio-maxillary conditions (33)	0.5 (7)	1 (7)	-0.5	0.5 (7)	0.4 (6)	0.1
ENT conditions (34)	1 (10)	2 (14)	-1	1 (10)	1 (11)	0
Psychiatric conditions (35)	9 (28)	1 (11)	8	9 (28)	8 (27)	1
Ophthalmological conditions(36)	1 (12)	4 (19)	-3	1 (12)	1 (11)	0
Diagnosis not classifiable (38)	26 (44)	21 (42)	5	26 (44)	28 (45)	-2
Nothing abnormal detected (39)	5 (22)	3 (17)	2	5 (22)	5 (22)	0
**N**	**16,338**	**5,345,363**		**16,338**	**75,359**	

[a] NHS accident and emergency codes; Data source: Hospital Episode Statistics A&E 2013/14; Government office region of attendance displayed instead of CCG area of A&E attended for brevity; standard deviations in parentheses

For peer review only

Table 2: Descriptive statistics: Annual counts of different types of hospital admission for no fixed abode and (matched) housed patients

	N	Mean (SD)	Min	Max	% of observations in range								
					0	1	2	3-5	6-9	10-19	20-29	30-49	50+
**Admissions**													
No fixed abode	63,604	0.47 (2.12)	0	127	84.01	7.69	3.23	3.25	1.08	0.58	0.09	0.04	0.03
Housed (matched)	295,037	0.23 (0.95)	0	100	87.44	8.03	2.38	1.69	0.34	0.11	0.01	<0.01	<0.01
Housed (before matching)	21,381,452	0.23 (0.85)	0	136	86.94	8.50	2.46	1.69	0.32	0.08	0.01	<0.01	<0.01
**Emergency admissions**													
No fixed abode	63,604	0.43 (2.07)	0	127	85.26	7.19	2.93	2.95	0.97	0.54	0.08	0.04	0.03
Housed (matched)	295,037	0.18 (0.86)	0	100	89.72	6.82	1.84	1.27	0.26	0.08	0.01	<0.01	<0.01
Housed (before matching)	21,381,452	0.17 (0.71)	0	131	89.99	6.86	1.76	1.13	0.20	0.05	<0.01	<0.01	<0.01
**Emergency admissions for any ACSC**													
No fixed abode	63,604	0.06 (0.65)	0	71	96.37	2.47	0.68	0.36	0.08	0.02	<0.01	<0.01	<0.01
Housed (matched)	295,037	0.03 (0.28)	0	22	97.59	1.90	0.32	0.16	0.03	0.01	<0.01	0	0
Housed (before matching)	21,381,452	0.04 (0.29)	0	44	97.26	2.14	0.39	0.19	0.03	0.01	<0.01	<0.01	0
**Emergency admissions for acute ACSC**													
No fixed abode	63,604	0.03 (0.25)	0	10	97.50	1.92	0.41	0.15	0.02	<0.01	0	0	0
Housed (matched)	295,037	0.02 (0.16)	0	10	98.52	1.29	0.13	0.05	<0.01	<0.01	0	0	0
Housed (before matching)	21,381,452	0.02 (0.17)	0	24	98.35	1.42	0.17	0.05	<0.01	<0.01	<0.01	0	0
**Emergency admissions for chronic ACSC**													
No fixed abode	63,604	0.03 (0.54)	0	61	98.53	0.95	0.28	0.17	0.05	0.01	<0.01	<0.01	0.01
Housed (matched)	295,037	0.02 (0.21)	0	22	98.93	0.80	0.16	0.09	0.02	0.01	<0.01	0	0
Housed (before matching)	21,381,452	0.02 (0.22)	0	44	98.73	0.95	0.19	0.10	0.02	<0.01	<0.01	<0.01	0

Data source: Hospital Episode Statistics admitted patient care 2014/15-2017/18; ACSC = ambulatory care-sensitive condition

For peer review only

Table 3: Negative binomial regression estimates (Incident Rate Ratios) of the effect of having no fixed abode on annual hospital admissions

	**Admissions**		**Emergency admissions for an ACSC**		
	**Any (1)**	**Emergency (2)**	**Any ACSC (3)**	**Acute (4)**	**Chronic (5)**
No fixed abode	1.82	2.12	1.70	1.81	1.52
	(0.05)	(0.07)	(0.08)	(0.07)	(0.10)
Year of admission					
2014 (reference category)					
2015	0.81	0.81	0.86	0.86	0.85
	(0.01)	(0.01)	(0.03)	(0.04)	(0.05)
2016	0.71	0.71	0.86	0.87	0.76
	(0.01)	(0.01)	(0.03)	(0.04)	(0.05)
2017	0.67	0.69	0.81	0.80	0.74
	(0.01)	(0.01)	(0.03)	(0.04)	(0.06)
Sex					
Female	1.29	1.08	1.09	1.19	0.95
	(0.04)	(0.04)	(0.06)	(0.06)	(0.08)
Age at time of attendance					
16 to 25 (reference category)					
26 to 35	1.19	1.24	1.38	1.59	1.09
	(0.05)	(0.06)	(0.11)	(0.11)	(0.15)
36 to 45	1.50	1.63	1.93	2.13	1.77
	(0.07)	(0.08)	(0.14)	(0.15)	(0.22)
46 to 55	1.86	2.00	2.54	2.23	3.22

For peer review only

	(0.09)	(0.11)	(0.19)	(0.17)	(0.40)
56 to 65	1.86	1.93	3.07	2.22	4.64
	(0.10)	(0.12)	(0.65)	(0.19)	(0.63)
66 to 75	3.07	3.38	7.21	4.55	11.67
	(0.43)	(0.49)	(1.60)	(0.55)	(3.34)
**N**	358,641	358,641	358,641	358,641	358,641

Data source: Hospital Episode Statistics admitted patient care 2014/15-2017/18; ACSC = ambulatory care-sensitive condition; standard errors in parentheses

For peer review only

Table 4: Count and percent of ambulatory care-sensitive emergency admissions for specific conditions

	Housed (% of total)	No fixed abode (% of total)
**Acute**		
Influenza, pneumonia, other vaccine preventable	837 (8%)	267 (6%)
Angina	87 (1%)	57 (1%)
Dehydration and gastroenteritis	788 (8%)	195 (5%)
Pyelonephritis and kidney/urinary tract infections	977 (10%)	184 (4%)
Perforated/bleeding ulcer	259 (3%)	82 (2%)
Cellulitis	1,098 (11%)	742 (18%)
Ear, nose and throat (ENT)	373 (4%)	52 (1%)
Dental	210 (2%)	71 (2%)
Convulsions and epilepsy	523 (5%)	501 (12%)
**Chronic**		
Chronic viral hepatitis B	5 (<1%)	0 (0%)
Diabetes	695 (7%)	376 (9%)
Anaemia	110 (1%)	38 (1%)
Epilepsy/dementia/atrial	1,435 (14%)	593 (14%)
Heart failure	296 (3%)	44 (1%)
Angina	402 (4%)	335 (8%)
Hypertension	61 (1%)	8 (<1%)
Bronchitis	1,123 (11%)	408 (10%)
Asthma	692 (7%)	157 (4%)
Total count of ACS emergency admissions	9,971	4,110
Number of patients	75,359	16,338

ACS = Ambulatory care-sensitive; Data source: Hospital Episode Statistics admitted patient care 2014/15-2017/18; percentages are rounded to the nearest whole number and may not add to 100

For peer review only

Figure 1: The effect of having no fixed abode on outcomes

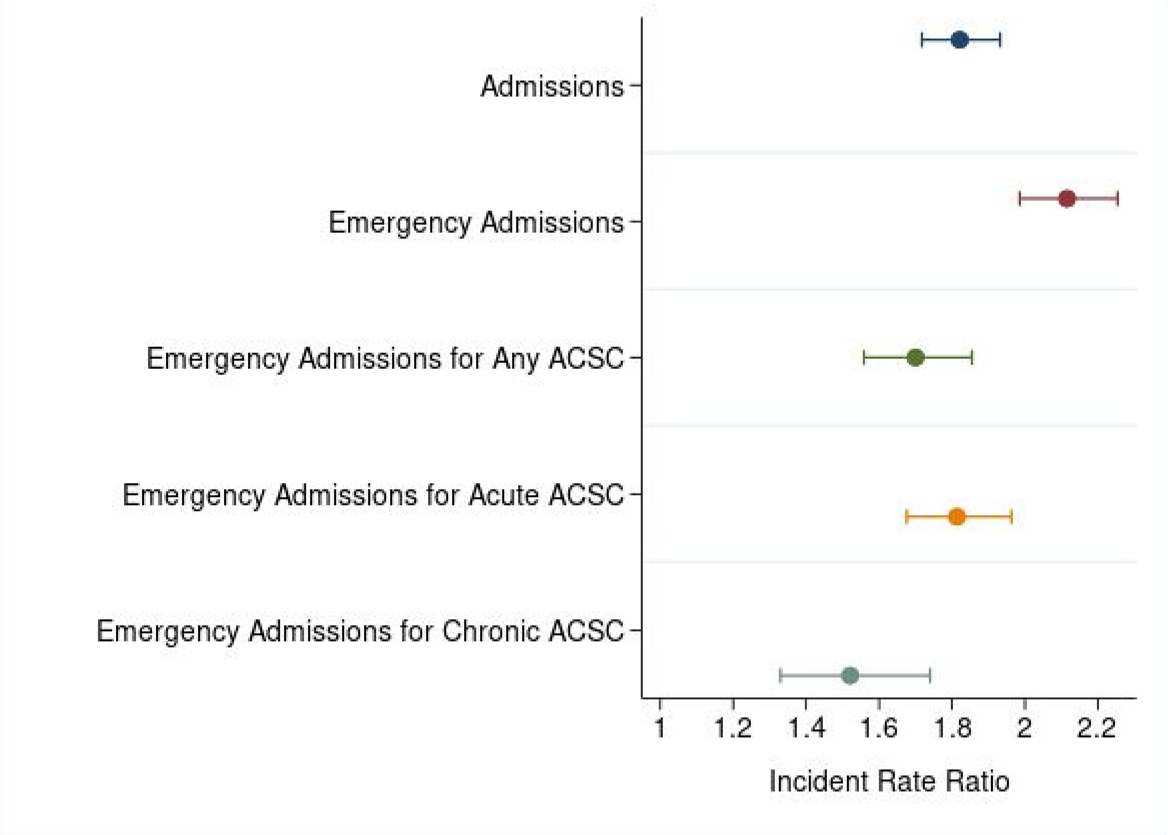

ACSC = Ambulatory care-sensitive condition; Data source: Hospital Episode Statistics admitted patient care 2014/15-2017/18; Full regression results in Table 2

Supplementary Material

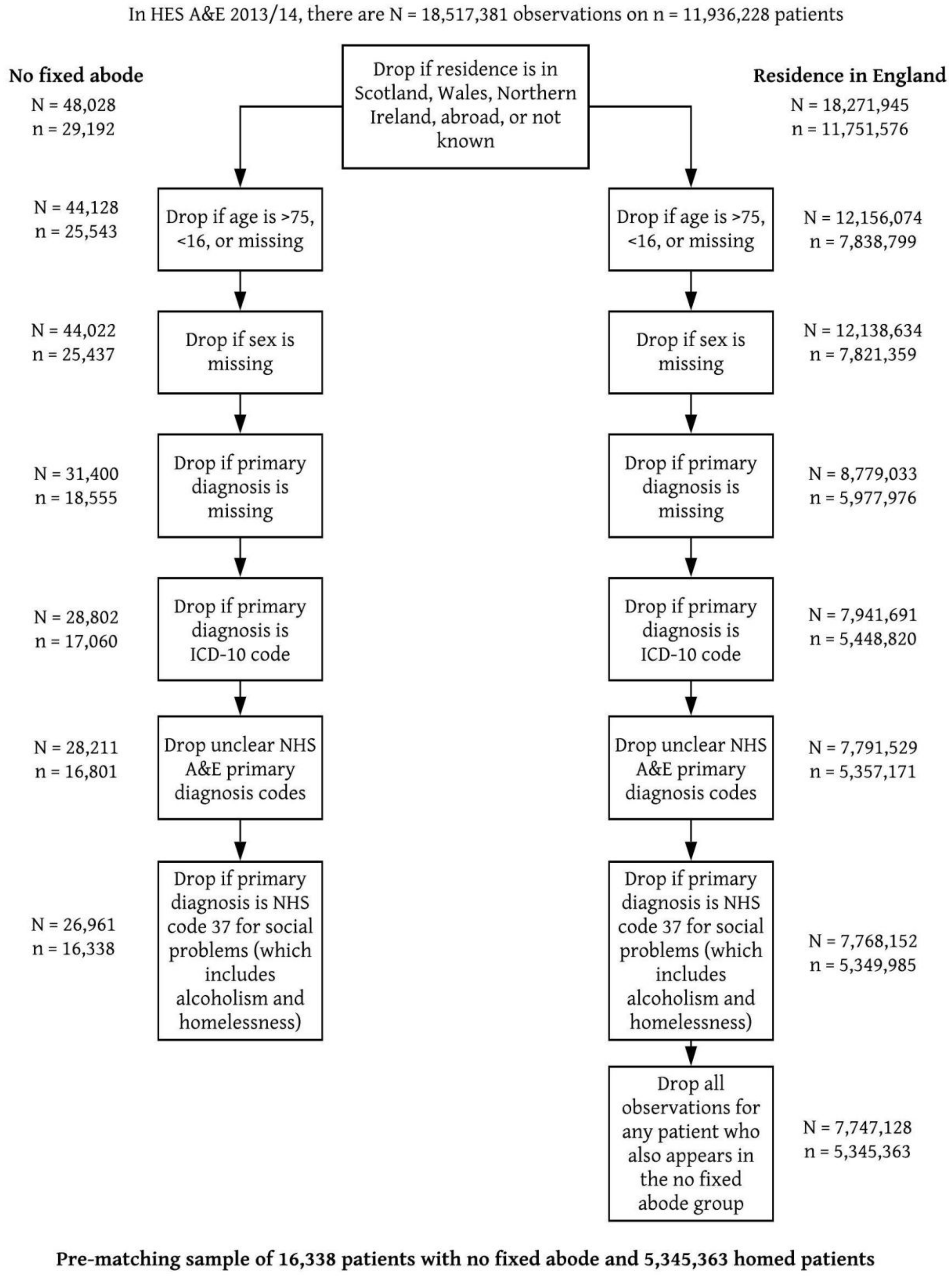

STROBE Statement—Checklist of items that should be included in reports of ***cohort studies***

	Item No	Recommendation	How we have included this
**Title and abstract**	1	(*a*) Indicate the study's design with a commonly used term in the title or the abstract	We use the term longitudinal in the title and explain our matching approach in the abstract.
		(*b*) Provide in the abstract an informative and balanced summary of what was done and what was found	Done
**Introduction**			
Background/rationale	2	Explain the scientific background and rationale for the investigation being reported	We have referenced studies of homeless persons' health and access to health care.
Objectives	3	State specific objectives, including any prespecified hypotheses	We state our objectives in the final paragraph of the introduction.
**Methods**			
Study design	4	Present key elements of study design early in the paper	We do this in the abstract and last paragraph of introduction.
Setting	5	Describe the setting, locations, and relevant dates, including periods of recruitment, exposure, follow-up, and data collection	Done, see methods section.
Participants	6	(*a*) Give the eligibility criteria, and the sources and methods of selection of participants. Describe methods of follow-up	Done, see methods section.
		(*b*) For matched studies, give matching criteria and number of exposed and unexposed	We have included a flow chart in the supplementary material.
Variables	7	Clearly define all outcomes, exposures, predictors, potential confounders, and effect modifiers. Give diagnostic criteria, if applicable	We have done this in the methods section.
Data sources/ measurement	8*	For each variable of interest, give sources of data and details of methods of assessment (measurement). Describe comparability of assessment methods if there is more than one group	Our one source of data is HES, which we have explained.
Bias	9	Describe any efforts to address potential sources of bias	We have described our use of deaths data.
Study size	10	Explain how the study size was arrived at	Done in flowchart and methods section.
Quantitative variables	11	Explain how quantitative variables were handled in the analyses. If applicable, describe which groupings were chosen and why	Done in methods section.
Statistical methods	12	(*a*) Describe all statistical methods, including those used to control for confounding	We described our matching and regression analysis.
		(*b*) Describe any methods used to examine subgroups and interactions	We didn't look at any subgroups within the two cohorts.

For peer review only - http://bmjopen.bmj.com/site/about/guidelines.xhtml

		(*c*) Explain how missing data were addressed	Done in methods section and flowchart.
		(*d*) If applicable, explain how loss to follow-up was addressed	N/A
		(*e*) Describe any sensitivity analyses	N/A
**Results**			
Participants	13*	(a) Report numbers of individuals at each stage of study—eg numbers potentially eligible, examined for eligibility, confirmed eligible, included in the study, completing follow-up, and analysed	See flowchart in supplementary material.
		(b) Give reasons for non-participation at each stage	Number of observations dropped at each stage is in flowchart, reason for dropping is explained in methods.
		(c) Consider use of a flow diagram	Included.
Descriptive data	14*	(a) Give characteristics of study participants (eg demographic, clinical, social) and information on exposures and potential confounders	We include demographic characteristics in Table 1, descriptive statistics for outcomes in Table 2.
		(b) Indicate number of participants with missing data for each variable of interest	We do not have any missing data for our outcomes. Number of observations dropped at each stage of sample selection are shown in flowchart.
		(c) Summarise follow-up time (eg, average and total amount)	Done, it is the same for every patient (4 financial years)
Outcome data	15*	Report numbers of outcome events or summary measures over time	Done, Table 2.
Main results	16	(*a*) Give unadjusted estimates and, if applicable, confounder-adjusted estimates and their precision (eg, 95% confidence interval). Make clear which confounders were adjusted for and why they were included	For each outcome, we include regression results with covariates. We include standard errors in the results table and confidence intervals in the abstract and main text.
		(*b*) Report category boundaries when continuous variables were categorized	We reported the age brackets that we used in the regressions.
		(*c*) If relevant, consider translating estimates of relative risk into absolute risk for a meaningful time period	N/A
Other analyses	17	Report other analyses done—eg analyses of subgroups and interactions, and sensitivity analyses	N/A
**Discussion**			
Key results	18	Summarise key results with reference to study objectives	Done in first section of discussion.

For peer review only - http://bmjopen.bmj.com/site/about/guidelines.xhtml

Limitations	19	Discuss limitations of the study, taking into account sources of potential bias or imprecision. Discuss both direction and magnitude of any potential bias	Done in limitations section.
Interpretation	20	Give a cautious overall interpretation of results considering objectives, limitations, multiplicity of analyses, results from similar studies, and other relevant evidence	Done in explanation section.
Generalisability	21	Discuss the generalisability (external validity) of the study results	Done in limitations section.
**Other information**			
Funding	22	Give the source of funding and the role of the funders for the present study and, if applicable, for the original study on which the present article is based	We have provided the sources of funding in acknowledgements.

*Give information separately for exposed and unexposed groups.

Note: An Explanation and Elaboration article discusses each checklist item and gives methodological background and published examples of transparent reporting. The STROBE checklist is best used in conjunction with this article (freely available on the Web sites of PLoS Medicine at http://www.plosmedicine.org/, Annals of Internal Medicine at http://www.annals.org/, and Epidemiology at http://www.epidem.com/). Information on the STROBE Initiative is available at http://www.strobe-statement.org.

For peer review only

BMJ Open

Comparative four year risk and type of hospital admission among homeless and housed emergency department attendees: longitudinal study of hospital records in England 2013-2018

Journal:	*BMJ Open*
Manuscript ID	bmjopen-2021-049811.R1
Article Type:	Original research
Date Submitted by the Author:	10-Jun-2021
Complete List of Authors:	Moss, Charlie; The University of Manchester, Health Organisation, Policy and Economics, Centre for Primary Care and Health Services Research, School of Health Sciences<br>Sutton, Matt; The University of Manchester, Health Organisation, Policy and Economics, Centre for Primary Care and Health Services Research, School of Health Sciences; The University of Melbourne, Melbourne Institute: Applied Economic and Social Research<br>Cheraghi-Sohi, Sudeh; The University of Manchester, NIHR Greater Manchester Patient Safety Translational Research Centre, Centre for Primary Care and Health Services Research, Manchester Academic Health Science Centre<br>Sanders, Caroline; The University of Manchester, NIHR Greater Manchester Patient Safety Translational Research Centre, Centre for Primary Care and Health Services Research, Manchester Academic Health Science Centre<br>Allen, Thomas; The University of Manchester, Manchester Centre for Health Economics, School of Health Sciences; University of Southern Denmark, Danish Centre for Health Economics, Department of Public Health
**Primary Subject Heading**:	Health services research
Secondary Subject Heading:	Epidemiology, Public health
Keywords:	ACCIDENT & EMERGENCY MEDICINE, PUBLIC HEALTH, SOCIAL MEDICINE

SCHOLARONE™
Manuscripts

I, the Submitting Author has the right to grant and does grant on behalf of all authors of the Work (as defined in the below author licence), an exclusive licence and/or a non-exclusive licence for contributions from authors who are: i) UK Crown employees; ii) where BMJ has agreed a CC-BY licence shall apply, and/or iii) in accordance with the terms applicable for US Federal Government officers or employees acting as part of their official duties; on a worldwide, perpetual, irrevocable, royalty-free basis to BMJ Publishing Group Ltd ("BMJ") its licensees and where the relevant Journal is co-owned by BMJ to the co-owners of the Journal, to publish the Work in this journal and any other BMJ products and to exploit all rights, as set out in our licence.

The Submitting Author accepts and understands that any supply made under these terms is made by BMJ to the Submitting Author unless you are acting as an employee on behalf of your employer or a postgraduate student of an affiliated institution which is paying any applicable article publishing charge ("APC") for Open Access articles. Where the Submitting Author wishes to make the Work available on an Open Access basis (and intends to pay the relevant APC), the terms of reuse of such Open Access shall be governed by a Creative Commons licence – details of these licences and which Creative Commons licence will apply to this Work are set out in our licence referred to above.

Other than as permitted in any relevant BMJ Author's Self Archiving Policies, I confirm this Work has not been accepted for publication elsewhere, is not being considered for publication elsewhere and does not duplicate material already published. I confirm all authors consent to publication of this Work and authorise the granting of this licence.

For peer review only

Comparative four year risk and type of hospital admission among homeless and housed emergency department attendees: longitudinal study of hospital records in England 2013-2018

Charlie Moss,[1] Matt Sutton,[1,2] Sudeh Cheraghi-Sohi,[3] Caroline Sanders,[3] Thomas Allen[4,5]

[1] Health Organisation, Policy and Economics (HOPE), Centre for Primary Care and Health Services Research, School of Health Sciences, The University of Manchester, Manchester, UK

[2] Melbourne Institute: Applied Economic and Social Research, The University of Melbourne, Melbourne, Australia

[3] NIHR Greater Manchester Patient Safety Translational Research Centre, Centre for Primary Care and Health Services Research, The University of Manchester, Manchester Academic Health Science Centre, Manchester, UK

[4] Manchester Centre for Health Economics, School of Health Sciences, The University of Manchester, Manchester, UK

[5] Danish Centre for Health Economics, Department of Public Health, The University of Southern Denmark, Odense, Denmark

Corresponding author: Charlie Moss, Suite 9, 6th floor, Williamson Building, Oxford Road, The University of Manchester, Manchester, M13 9PL, UK

Email: charlie.moss@manchester.ac.uk

Keywords: Homelessness; Health service use; Emergency health care; Health care access

For peer review only - http://bmjopen.bmj.com/site/about/guidelines.xhtml

DECLARATIONS

Ethics approval and consent to participate

Not applicable

Consent for publication

Not applicable

Availability of data and materials

The Hospital Episode Statistics data that support the findings of this study are available from NHS Digital but restrictions apply to the availability of these data, which were used under license for the current study, and so are not publicly available.

Competing interests

The authors declare that they have no competing interests.

Funding

This research was funded jointly by a NIHR Research Methods Fellowship [RM-FI-2017-08-018] and the NIHR Greater Manchester Patient Safety Translational Research Centre [PSTRC-2016-003]. The views expressed are those of the authors and not necessarily those of the NIHR or the Department of Health and Social Care.

Patient and public involvement

Patients or the public were not involved in the design of this study.

For peer review only

For peer review only - http://bmjopen.bmj.com/site/about/guidelines.xhtml

ABSTRACT

Objectives: People experiencing homelessness are frequent users of secondary care. Currently, there is no study of potentially preventable admissions for homeless patients in England. We aim to estimate the number of potentially preventable hospital admissions for homeless patients and compare to housed patients with similar characteristics.

Design: Retrospective matched cohort study

Setting: Hospitals in England.

Participants: 16,161 homeless patients and 74,780 housed patients aged 16 to 75 who attended an emergency department (ED) in England in 2013/14, matched on the basis of age, sex, ED attended, and primary diagnosis.

Primary and secondary outcome measures: Annual counts of admissions, emergency admissions, ambulatory care-sensitive (ACS) emergency admissions, acute ACS emergency admissions, and chronic ACS emergency admissions over the following four years (2014/15 to 2017/18). We additionally compare the prevalence of specific ACS conditions for homeless and housed patients.

Results: Mean admissions per 1000 patients per year were 470 for homeless patients and 230 for housed patients. Adjusted for confounders, annual admissions were 1.79 times higher (IRR=1.79; 95% CI [1.69 to 1.90]), emergency admissions 2.08 times higher (IRR=2.08; 95% CI [1.95 to 2.21]), and ACS admissions 1.65 times higher (IRR=1.65; 95% CI [1.51 to 1.80]), compared with housed patients. The effect was greater for acute (IRR=1.78; 95% CI [1.64 to 1.93]) than chronic (IRR=1.45; 95% CI [1.27 to 1.66]) ACS conditions. ACS conditions that were relatively more common for homeless patients were cellulitis, convulsions/epilepsy, and chronic angina.

Conclusions: Homeless patients use hospital services at higher rates than housed patients, particularly emergency admissions. ACS admissions of homeless patients are higher which suggests some admissions may be potentially preventable with improved access to primary care. However, these admissions comprise a small share of total admissions.

For peer review only - http://bmjopen.bmj.com/site/about/guidelines.xhtml

For peer review only

Strengths and limitations of this study

- Large sample of 90,941 patients
- Comparative study design
- Use of linked mortality data to avoid immortality bias
- "No fixed abode" flag used to identify patients who are homeless is not a perfect indicator of homelessness
- Use of emergency department attendances data to identify study participants may affect generalisability to less-regular emergency department attendees

For peer review only

Background

Homelessness remains a problem in England, as in many developed countries.[1,2] The homelessness charity, Shelter, estimates that 307,000 people experienced homelessness in England in 2017,[3] although this is considered an underestimate as it does not count the vulnerably housed, or those missed by official statistics who often rely on friends and family.

Homelessness is associated with poor health and a high rate of mortality.[4–6] Homeless people often have comorbidities usually found in substantially older people.[1,7] Tri-morbidity,[8] is a term used to describe the common combination of physical ill-health, mental ill-health and drug or alcohol misuse which gives rise to complex needs. In 2018, the average age of death for homeless men and women in England and Wales was 45 and 43 years, respectively, compared with 76 and 81 years for the general population.[9] A 2019 study estimated that 30% of deaths amongst homeless people are from conditions amenable to health care, compared to 23% of deaths in a comparison group of housed people from the most deprived quintile of areas in England.[10]

There is some evidence that homeless people have limited access to routine health care. Homeless people face barriers to registering with and accessing mainstream primary care services,[11,12] often due to organisational barriers (e.g. inflexible appointment schedules) and previous negative experiences, including experience (or fear) of stigma when attempting to access services. People who are homeless are less likely to be registered with a GP than the general population.[13] It is possible that poor access to primary care contributes to the high use of emergency departments and inpatient care by people who are homeless,[7,14,15] which is both costly and lacking in continuity of care for patients who often have complex health problems. The latter is viewed to be especially important for meeting long-term needs and delivering care with compassion for homeless people who have common experiences of trauma.[16]

It has been proposed that emergency hospital admissions related to some conditions, known as ambulatory care-sensitive (ACS) conditions, could be avoided if timely and effective primary care was provided.[17,18] Diabetes, influenza, asthma, and anaemia are examples of such conditions. Elevated ACS hospital admissions may also provide indirect insights into the quality of primary care provided, although there are many aspects of quality of care that they will fail to capture.[19] One study has suggested that there were 1.77 million ACS admissions in England during 2011-2012, accounting for 10.9 million bed days.[20]

For peer review only - http://bmjopen.bmj.com/site/about/guidelines.xhtml

Currently, there is no study of potentially preventable admissions for homeless patients in England. We add to the evidence by using a comparative design: we use national data to match a cohort of homeless patients to a cohort of housed patients with similar observable characteristics. We compare the relative frequency of hospital admissions for each cohort in the following four years, and the prevalence of specific ACS conditions. We then discuss the extent to which these admissions may be preventable with improved access to primary care.

Methods

In England, administrative data are collected on National Health Service (NHS) hospital service use for all admissions, accident and emergency (A&E) attendances and outpatient appointments.[21] These Hospital Episode Statistics (HES) data contain information on diagnoses and treatment, as well as provider and patient characteristics. Each patient has a unique anonymised identifier which allows individual patients to be tracked when they are treated or seen in different hospital settings and over time.

Sample definition

We used HES A&E attendances data to identify two groups of patients: housed and homeless. Within HES there are fields derived from the patient's postcode which assign patients to different administrative geographies. We used the field for local authority district of residence, where those patients without a postcode are coded "no fixed abode".

All patients are registered when they arrive at the emergency department. They are asked for their name, date of birth and address. Whether they are homeless or housed does not affect their entitlement to treatment and there is no direct incentive for patients to not disclose their true status. The emergency department needs the address details to know which health authority to charge for the care episode. They therefore have an incentive to record patients' residential locations accurately as this is needed for billing.

Although there is not a study of the validity of the no fixed abode indicator, the age and sex profile of the patients included in this study who were recorded as having no fixed abode (mean age 38, 76% male) is similar to other studies of people experiencing homelessness in England.[13,22–24]

For peer review only

We defined our sample of homeless patients as those who had an A&E attendance in the 2013/14 financial year and were recorded as having "no fixed abode" in the above field. The housed sample were those whose recorded local authority indicated that they resided in England.

For each A&E attendance in 2013/14, we retained the following variables required to match homeless and housed patients: sex, age, primary diagnosis and the emergency department the patient attended.

We restricted our analysis to patients aged 16 to 75. We removed observations with missing or poor quality data in the fields used for matching, such as if sex was recorded as not specified or not known. Where present, diagnosis codes are a mixture of NHS A&E diagnosis condition codes[25] (92% amongst observations with "no fixed abode") and ICD-10 codes.[26]

We retained only the observations with NHS A&E diagnosis codes and matched using only the first two characters, corresponding to 39 broad diagnosis groups such as "head injury", "cardiac conditions", and "respiratory conditions". We excluded observations where the first two characters were not in the valid range of 01 to 39 (4% of observations with "no fixed abode"). These may have been intended to be recorded with a code group between 04 and 09, but were mistakenly coded without the preceding zero. We were unable to distinguish these errors from other coding errors.

Finally, we excluded those patients who had a primary diagnosis for "social problems" (NHS Accident and Emergency diagnosis code group 37; 3% of observations with "no fixed abode") in order to ensure that homeless patients were identified exclusively on the basis of their area of residence. This prevented homeless patients populating the 'housed' group via this diagnosis code.

We include a flowchart of observations removed at each stage in the supplementary material.

After the above exclusions, two mutually exclusive groups of patients remained: (1) 5,345,363 patients recorded as housed in England on all of their A&E attendances during 2013/14 and (2) 16,338 patients recorded as having no fixed abode on at least one of their A&E attendances during 2013/14.

Matching of homeless with housed patients

For peer review only - http://bmjopen.bmj.com/site/about/guidelines.xhtml

We used variable ratio matching[27] to match each of the 16,338 homeless patients with up to five housed patients on combinations of sex, single year of age, primary diagnosis and the emergency department the patient attended.

After matching, the two cohorts comprised 16,161 homeless patients and 74,780 housed patients.

Outcomes

We used each matched patient's unique identifier to locate their admissions in the HES data for the following four financial years (2014/15, 2015/16, 2016/17, 2017/18).

We used two outcomes from the CCG Outcomes Indicator Set (CCG OIS)[28] to identify which emergency admissions were for an ACS condition: unplanned hospitalisation for chronic ACS conditions (indicator 2.6) and emergency admission for acute conditions that should not usually require a hospital admission (indicator 3.1).

We constructed five counts for each patient in each financial year: admissions; emergency admissions; emergency admissions for any ACS condition; emergency admissions for an acute ACS condition; emergency admissions for a chronic ACS condition.

If a patient did not appear in the admissions data, we assigned a count of zero admissions for that financial year. Some patients died within the study period, and mortality is likely to differ between the housed and homeless cohorts. We therefore corrected this source of bias by using linked death records[29] to remove patients who die from the sample in the years following their death.

Using pooled data for all four years, we estimated negative binomial regression models for each outcome due to over-dispersion. We present effects as Incident Rate Ratios (IRRs) and calculated marginal effects for the key variables to show the magnitude of these effects on the original scale. We used weights because each homeless patient is matched to between one and five housed patients. Each homeless patient has a weight of 1 and the weights assigned to housed patients are the reciprocal of the number of housed patients matched to the homeless patient.

For each outcome, we estimated a model which included a binary indicator for whether the patient was recorded as homeless in 2013/14, year indicators, and indicators for the matching characteristics: sex, A&E attended in 2013/14, primary diagnosis group at A&E attendance in

For peer review only - http://bmjopen.bmj.com/site/about/guidelines.xhtml

2013/14, and ten-year age categories to allow for an expected non-linear relationship between age and all outcomes. We used robust standard errors clustered at the individual level because the sample includes multiple observations for each individual.

Results

Matching of homeless with housed patients

We were able to match 16,161 out of 16,338 homeless patients to at least one housed patient. Prior to matching, the homeless group were younger, more likely to be male and were more likely to attend A&E in a London hospital (Table 1). Whilst attendances were distributed relatively similarly across the two populations in terms of primary diagnoses, there were a few notable exceptions. First, poisonings (which include overdoses) comprised a higher share of attendances in homeless patients compared with housed: a homeless patient's attendance was for a diagnosis of poisoning 14 times more often. Second, homeless patient's attendances were for psychiatric conditions 9 times more often.

Conversely, acute injuries (such as laceration, sprain, dislocation and contusion) were typically more likely for housed patients compared with homeless patients. After matching, the two groups were markedly more balanced in terms of their characteristics.

Outcomes

The mean values of all outcomes were greater for homeless patients than for housed patients, both before and after matching (Table 2).

Each year, most people in the homeless and housed cohorts did not have any admissions. However, for all outcomes, patients in the homeless cohort more frequently had at least one admission each year, in comparison with the housed patients, both before and after matching.

A markedly greater percentage of the homeless cohort had a very large number of admissions and emergency admissions per year, compared with the housed cohort; 0.73% of the homeless cohort had ten or more admissions each year, compared with 0.12% of the matched

For peer review only - http://bmjopen.bmj.com/site/about/guidelines.xhtml

For peer review only

housed cohort, and 0.68% of the homeless cohort had ten or more emergency admissions each year, compared with 0.1% of the housed cohort.

Table 3 presents the estimates from the negative binomial regression models. When the covariates are matched across groups, the homeless cohort had a higher count of all outcomes (Table 3; Figure 1). For homeless patients, annual admissions were 1.79 times higher (IRR=1.79; 95% confidence interval (CI) [1.69 to 1.90]), emergency admissions 2.08 times higher (IRR=2.08; 95% CI [1.95 to 2.21]), and ACS admissions 1.65 times higher (IRR=1.65; 95% CI [1.51 to 1.80]), compared with housed patients. The effect was greater for acute (IRR=1.78; 95% CI [1.64 to 1.93]) than chronic (IRR=1.45; 95% CI [1.27 to 1.66]) ACS conditions.

The largest marginal effect of homelessness was on emergency admissions; the homeless cohort had 0.225 additional emergency admissions (95% CI [0.198 to 0.252]) and 0.025 additional ACS admissions (95% [CI 0.019 to 0.03]) each year compared with the housed cohort.

Admissions for several specific ACS conditions were relatively more common for the homeless cohort than for the housed cohort (Table 4). Of the acute ACS conditions, the percentage of total homeless ACS admissions that were for cellulitis was 1.5 times that of the housed cohort (18% compared with 12%), and the percentage for convulsions and epilepsy was 2.4 times that of the housed cohort (12% compared with 5%). Of the chronic ACS conditions, the percentage of homeless ACS admissions for angina was twice that of the housed cohort (8% compared with 4%).

For peer review only

Discussion

Summary of main findings

Homeless patients had more admissions in general, more emergency admissions and more admissions for an ACS condition than housed patients with similar characteristics. This effect was greater for acute ACS conditions than for chronic ACS conditions.

We estimated that, compared to 1,000 housed patients with similar characteristics, 1,000 homeless patients would collectively have 225 more emergency admissions per year, and 25

For peer review only - http://bmjopen.bmj.com/site/about/guidelines.xhtml

more ACS emergency admissions per year. This suggests that ACS admissions account for around 11% of the additional emergency admissions experienced by homeless patients.

Acute ACS conditions for which emergency admission was more common for the homeless cohort were cellulitis and convulsions/epilepsy, and the one chronic ACS condition was angina.

Explanation of notable findings

Emergency admission for chronic angina was twice as common in the homeless cohort compared to the housed cohort. This could be due to the relatively high prevalence of cardio-vascular disease (CVD) in the homeless population,[6] and/or poor subsequent management of CVD in homeless patients compared with housed patients.

The higher number of acute ACS admissions for homeless patients is driven partly by emergency admissions for cellulitis in particular, and the causes of this are likely to be complex. Hospital admissions related to cellulitis are common amongst individuals who inject illicit drugs,[30,31] and homelessness is more common amongst individuals with problematic substance misuse.[22] The differential prevalence of cellulitis in the homeless cohort may therefore be indicative of increased rates of drugs misuse. This may also be tentatively indicated by the higher prevalence of admissions for convulsions, and the higher prevalence of A&E attendances for poisonings in the homeless cohort. Cellulitis is also strongly associated with comorbidity and conditions such as diabetes, however the causal relationship is likely to be bi-directional.[30] Furthermore, cellulitis is difficult to diagnose in primary care and even if patients attend primary care, these difficulties may lead to delays in treatment and subsequent and appropriate use of emergency healthcare.[32]

There is likely to be a complex interplay of factors at multiple levels which lead to the differences observed. People experiencing homelessness face barriers to accessing healthcare at the macro-level, such as potential disincentives for providers to provide more accessible services.[15] At the meso-level, organisational factors such as where, how and when appointments are offered in primary care place further barriers to care.[11,12] Finally, at the micro-level, both patient and professional factors are relevant. People experiencing homelessness have been described as having chaotic lifestyles.[15] In combination with the perceived and/or experienced stigma and discrimination from health professionals, this may lead to poor or delayed engagement with the health service. These social and structural issues

For peer review only - http://bmjopen.bmj.com/site/about/guidelines.xhtml

mean that health problems can degenerate into more serious problems that subsequently require contact with emergency services.

Comparison with existing research

A study of homeless patients admitted to hospital in California in 2010 found that the odds that the admission was ACS was increased when the patient was black, admitted from the emergency department or transferred from another health care facility, or did not have Medicare.[33] A study of hospital inpatient records in Ireland from 2005-2014 found that of 2,051 emergency admissions for patients with "no fixed abode", 280 (13.7%) were for an ACS condition. The most common ACS conditions amongst the admissions were convulsions/epilepsy (32.9%), cellulitis (22.1%) and COPD (10.4%).[34] Neither study compared ACS admissions of homeless patients with those of housed patients.

We add to the current evidence by using a comparative design: we matched a group of homeless patients to a group of housed patients with similar observable characteristics. We also used more recent data so our analysis is reflective of factors which have changed in the 2010s such as economic policies enacted by the UK government and the increase in homelessness in England.[23]

Limitations

The no fixed abode flag in HES is not a perfect indicator of homelessness. Some patients are recorded as having no fixed abode on one or more attendances and as being homed on other attendances. In part this will be the result of genuine changes in housing status, but we also suspect that (although there is no direct incentive for them to do so) false information may be provided in some cases, such as the address of a friend or relative. This may bias our estimates towards the null.

Several factors may affect the generalisability of our study. First, it is possible that patients recorded as having no fixed abode are some of the most vulnerable and isolated homeless people who do not have an address to give. Second, because there is no comprehensive database of all people experiencing homelessness, we selected patients to include in the study on the basis of A&E attendances. These patients may be more likely to be admitted in an emergency than less regular A&E attendees, and the size of this difference may vary across the two cohorts.

For peer review only - http://bmjopen.bmj.com/site/about/guidelines.xhtml

We used linked death records to remove patients who died from the sample in the years following the year in which they died. The mortality rates were 5.06% in the homeless cohort and 3.76% in the housed cohort within the study period. We included the count of admissions during a financial year when the patient died part way through the financial year. This biases the difference in admission rates between homeless and housed cohorts downwards due to differences in exposure time.

We matched homeless patients to housed patients on the basis of sex, age, emergency department attended in 2013/14, and the diagnosis received on that attendance. Matching on this combination of variables allowed us to match 16,161 homeless patients (out of the total possible 16,338) to at least one housed patient. However, as with all matching studies, there is a threat of residual confounding.

Implications for policy and research

Dixon-Woods et al. have previously drawn attention to the complex interplay of factors (perceptions of need, priorities, attitudes of staff, resources, policies) that create inequalities and vulnerabilities for marginalised and disadvantaged groups in terms of access to healthcare.[35] Additional research has highlighted this complex interplay of barriers regarding access to primary care that can be viewed as creating a two-tier system for marginalised or 'hard-to-reach' groups.[36,37]

Our results suggest that patients who have experienced homelessness are admitted to hospital more frequently than housed patients for conditions classified as suitable for treatment and management in a primary care setting. Addressing structural factors such as improved access to primary care could potentially reduce some of these admissions. However, the fact that only 11% of the additional emergency admissions experienced by the homeless cohort were ACS suggests that other approaches are needed if the goal is to reduce homeless persons' admissions to hospital. Our results may suggest that interventions which strengthen support for homeless A&E attendees have the potential to reduce future hospital admissions.

Additionally, the likely relevance of other micro-level factors as a driver of some of the ACS admissions is a reminder of the complexity of health problems experienced by people who are homeless, which often have causes that are outside of the control of the healthcare system. The link between ACS admissions and access to primary care is less clear for this population than it might be for others.

For peer review only - http://bmjopen.bmj.com/site/about/guidelines.xhtml

Authors' contributions

SCS and CS conceived the original idea. MS and TA designed the empirical approach. CM carried out data analysis and drafted the first version of the manuscript. All authors contributed heavily to interpretation of results and editing the manuscript for the final version.

References

1. Fazel, S., Geddes, J. R. & Kushel, M. The health of homeless people in high-income countries: descriptive epidemiology, health consequences, and clinical and policy recommendations. *The Lancet* **384**, 1529–1540 (2014).
2. The Lancet. A shared future for all: let's talk about homelessness. *The Lancet* **391**, 179 (2018).
3. Shelter. *Far from alone: homelessness in Britain in 2017*. https://assets.ctfassets.net/6sxvmndnpn0s/6c6fuC7K5H9jFqZEbI4LLs/3be8d1e3086518c46cf048873e5aa3f6/8112017_Far_From_Alone.pdf (2017).
4. Morrison, D. S. Homelessness as an independent risk factor for mortality: results from a retrospective cohort study. *Int. J. Epidemiol.* **38**, 877–883 (2009).
5. Aldridge, R. W. *et al.* Morbidity and mortality in homeless individuals, prisoners, sex workers, and individuals with substance use disorders in high-income countries: a systematic review and meta-analysis. *The Lancet* **391**, 241–250 (2018).
6. Nanjo, A. *et al.* Prevalence, incidence, and outcomes across cardiovascular diseases in homeless individuals using national linked electronic health records. *Eur. Heart J.* **41**, 4011–4020 (2020).

For peer review only - http://bmjopen.bmj.com/site/about/guidelines.xhtml

7. Bowen, M. *et al.* Multimorbidity and emergency department visits by a homeless population: a database study in specialist general practice. *Br. J. Gen. Pract.* **69**, e515–e525 (2019).
8. Pathway. Homeless and inclusion health standards for commissioners and service providers. (2018).
9. Office for National Statistics. *Deaths of homeless people in England and Wales: 2018*. https://www.ons.gov.uk/peoplepopulationandcommunity/birthsdeathsandmarriages/deaths/bulletins/deathsofhomelesspeopleinenglandandwales/2018 (2019).
10. Aldridge, R. W. *et al.* Causes of death among homeless people: a population-based cross-sectional study of linked hospitalisation and mortality data in England. *Wellcome Open Res.* **4**, 49 (2019).
11. Rae, B. E. & Rees, S. The perceptions of homeless people regarding their healthcare needs and experiences of receiving health care. *J. Adv. Nurs.* **71**, 2096–2107 (2015).
12. Gunner, E. *et al.* Provision and accessibility of primary healthcare services for people who are homeless: a qualitative study of patient perspectives in the UK. *Br. J. Gen. Pract.* **69**, e526–e536 (2019).
13. Elwell-Sutton, T., Fok, J., Albanese, F., Mathie, H. & Holland, R. Factors associated with access to care and healthcare utilization in the homeless population of England. *J. Public Health* fdw008 (2016) doi:10.1093/pubmed/fdw008.
14. Hewett, N. *Closing the gap through changing relationships*. https://www.health.org.uk/sites/default/files/PromotingCompassionateHealthcareHomelessPeople_report.pdf (2013).
15. Office of the Chief Analyst. Healthcare for single homeless people. (2010).
16. Pathway *et al.* Psychologically informed services for homeless people: good practice guide. (2012).

For peer review only - http://bmjopen.bmj.com/site/about/guidelines.xhtml

17. Billings, J. *et al.* Impact Of Socioeconomic Status On Hospital Use In New York City. *Health Aff. (Millwood)* **12**, 162–173 (1993).

18. Sanderson, C. & Dixon, J. Conditions for Which Onset or Hospital Admission is Potentially Preventable by Timely and Effective Ambulatory Care. *J. Health Serv. Res. Policy* **5**, 222–230 (2000).

19. Hodgson, K., Deeny, S. R. & Steventon, A. Ambulatory care-sensitive conditions: their potential uses and limitations. *BMJ Qual. Saf.* **28**, 429–433 (2019).

20. Busby, J., Purdy, S. & Hollingworth, W. How do population, general practice and hospital factors influence ambulatory care sensitive admissions: a cross sectional study. *BMC Fam. Pract.* **18**, (2017).

21. NHS Digital. Hospital Episode Statistics (HES). https://digital.nhs.uk/data-and-information/data-tools-and-services/data-services/hospital-episode-statistics (2021).

22. Bramley, G. *et al. Hard edges: mapping severe and multiple disadvantage*. (2015).

23. Office for National Statistics. UK homelessness: 2005 to 2018. https://www.ons.gov.uk/peoplepopulationandcommunity/housing/articles/ukhomelessness/2005to2018 (2019).

24. St Mungo's. *CHAIN annual report: street to home*. 20–21 https://www.mungos.org/app/uploads/2017/07/chain_street_to_home_annual_report_2013-14.pdf (2013).

25. NHS Digital. HES data dictionary: accident and emergency. 47 (2020).

26. World Health Organisation. International classification of diseases and related health problems: 10th revision. (2016).

27. Stuart, E. A. Matching Methods for Causal Inference: A Review and a Look Forward. *Stat. Sci.* **25**, (2010).

For peer review only - http://bmjopen.bmj.com/site/about/guidelines.xhtml

28. NHS Digital. Clinical commissioning group outcomes indicator set (CCG OIS). https://digital.nhs.uk/data-and-information/publications/statistical/ccg-outcomes-indicator-set (2020).

29. Office for National Statistics. Deaths. https://www.ons.gov.uk/peoplepopulationandcommunity/birthsdeathsandmarriages/deaths#datasets.

30. Wright, T. *et al.* Prevalence and severity of abscesses and cellulitis, and their associations with other health outcomes, in a community-based study of people who inject drugs in London, UK. *PLOS ONE* **15**, e0235350 (2020).

31. Marks, M. *et al.* Needles and the damage done: Reasons for admission and financial costs associated with injecting drug use in a Central London Teaching Hospital. *J. Infect.* **66**, 95–102 (2013).

32. Patel, M. *et al.* An interview study to determine the experiences of cellulitis diagnosis amongst health care professionals in the UK. *BMJ Open* **10**, e034692 (2020).

33. White, B. M., Ellis Jr, C. & Simpson, K. N. Preventable hospital admissions among the homeless in California: A retrospective analysis of care for ambulatory care sensitive conditions. *BMC Health Serv. Res.* **14**, (2014).

34. O'Farrell, A., Evans, D. S. & Allen, M. The Epidemiology of Emergency In-Patient Hospitalisations Among Those with 'No Fixed Abode' (Homeless) 2005-2014: What Lessons Can Be Learnt. *Ir. Med. J.* **109**, 464 (2016).

35. Dixon-Woods, M. *et al.* Conducting a critical interpretive synthesis of the literature on access to healthcare by vulnerable groups. *BMC Med. Res. Methodol.* **6**, (2006).

36. Bristow, K. *et al.* Help Seeking and Access to Primary Care for People from "Hard-to-Reach" Groups with Common Mental Health Problems. *Int. J. Fam. Med.* **2011**, 1–10 (2011).

For peer review only - http://bmjopen.bmj.com/site/about/guidelines.xhtml

37. O'Donnell, P., Tierney, E., O'Carroll, A., Nurse, D. & MacFarlane, A. Exploring levers and barriers to accessing primary care for marginalised groups and identifying their priorities for primary care provision: a participatory learning and action research study. *Int. J. Equity Health* **15**, (2016).

For peer review only

For peer review only - http://bmjopen.bmj.com/site/about/guidelines.xhtml

Table 1: Characteristics of homeless and housed groups before and after matching

	Before matching			After matching		
	Homeless	Housed	Difference	Homeless	Housed	Difference
Individual characteristics						
Female (%)	24	49	-25	24	24	0
Age (years) [S.D]	38.02 [13.66]	41.45 [16.84]	-3.43	37.95 [13.66]	37.65 [13.66]	0.3
Government office region of attendance (%)						
East	13	9	4	13	13	0
East Midlands	3	8	-5	3	3	0
London	31	16	15	31	30	1
North East	1	4	-3	1	1	0
North West	21	19	2	21	22	-1
South East	16	13	3	16	16	0
South West	5	9	-4	4	4	0
West Midlands	5	9	-4	5	5	0
Yorkshire & the Humber	5	12	-7	5	6	-1
***Primary diagnosis group code* (%)** [a]						
Laceration (01)	5	6	-1	5	6	-1
Contusion/abrasion (02)	3	4	-1	3	3	0
Soft tissue inflammation (03)	2	6	-4	2	3	-1
Head injury (04)	4	3	1	4	5	-1
Dislocation/fracture/joint injury/amputation (05)	4	7	-3	4	4	0
Sprain/ligament injury (06)	3	7	-4	3	3	0
Muscle/tendon injury (07)	2	3	-1	2	2	0
Nerve injury (08)	0.1	0.2	-0.1	0.1	0.1	0
Vascular injury (09)	0.1	0.1	0	0.1	0.03	0.07
Burns and scalds (10)	0.4	1	-0.6	0.4	0.3	0.1
Electric shock (11)	0.02	0.1	-0.08	0.02	0.03	-0.01
Foreign body (12)	0.5	1	-0.5	0.5	0.5	0

For peer review only

For peer review only - http://bmjopen.bmj.com/site/about/guidelines.xhtml

Bites/stings (13)	0.3	1	-0.7	0.3	0.3	0
Poisoning (inc. overdose) (14)	14	1	13	14	12	2
Near drowning (15)	0.01	0.01	0	0.01	0.003	0.007
Visceral injury (16)	0.1	0.05	0.05	0.05	0.03	0.02
Infectious disease (17)	1	1	0	1	1	0
Local infection (18)	2	2	0	2	2	0
Septicaemia (19)	0.1	0.2	-0.1	0.1	0.05	0.05
Cardiac conditions (20)	2	4	-2	2	3	-1
Cerebro-vascular conditions (21)	0.3	1	-0.7	0.3	0.3	0
Other vascular conditions (22)	0.5	1	-0.5	0.5	0.3	0.2
Haematological conditions (23)	0.2	0.3	-0.1	0.1	0.1	0.1
Central nervous system conditions (exc. Strokes) (24)	3	2	1	3	3	0
Respiratory conditions (25)	2	3	-1	2	2	0
Gastrointestinal conditions (26)	4	6	-2	4	5	-1
Urological conditions (inc. cystitis) (27)	1	2	-1	1	1	0
Obstetric conditions (28)	0.2	1	-0.8	0.2	0.2	0
Gynaecological conditions (29)	0.5	2	-1.5	1	1	0
Diabetes and other endocrinological conditions (30)	0.3	0.4	-0.1	0.3	0.2	0.1
Dermatological conditions (31)	0.5	1	-0.5	0.5	0.4	0.1
Allergy (inc. anaphylaxis) (32)	0.2	1	-0.8	0.2	0.1	0.1
Facio-maxillary conditions (33)	0.5	1	-0.5	0.5	0.4	0.1
ENT conditions (34)	1	2	-1	1	1	0
Psychiatric conditions (35)	9	1	8	9	8	1
Ophthalmological conditions(36)	1	4	-3	1	1	0
Diagnosis not classifiable (38)	26	21	5	26	29	-3
Nothing abnormal detected (39)	5	3	2	5	5	0
**N**	**16,338**	**5,345,363**		**16,161**	**74,780**	

[a] NHS accident and emergency codes; Data source: Hospital Episode Statistics A&E 2013/14; Government office region of attendance displayed instead of A&E attended for brevity

For peer review only - http://bmjopen.bmj.com/site/about/guidelines.xhtml

Table 2: Descriptive statistics: Annual counts of different types of hospital admission for homeless and housed patients

	n	Mean (SD)	Min	Max	% of observations in range								
					0	1	2	3-5	6-9	10-19	20-29	30-49	50+
**Admissions**													
Homeless (matched)	62,910	0.47 (2.12)	0	127	84.06	7.68	3.22	3.24	1.07	0.57	0.09	0.04	0.03
Housed (matched)	292,842	0.23 (0.94)	0	84	87.34	8.10	2.35	1.72	0.36	0.12	0.01	<0.01	<0.01
Housed (before matching)	21,381,452	0.23 (0.85)	0	136	86.94	8.50	2.46	1.69	0.32	0.08	0.01	<0.01	<0.01
**Emergency admissions**													
Homeless (matched)	62,910	0.43 (2.08)	0	127	85.31	7.17	2.92	2.95	0.97	0.53	0.08	0.04	0.03
Housed (matched)	292,842	0.18 (0.83)	0	76	89.61	6.92	1.82	1.28	0.27	0.09	0.01	<0.01	<0.01
Housed (before matching)	21,381,452	0.17 (0.71)	0	131	89.99	6.86	1.76	1.13	0.20	0.05	<0.01	<0.01	<0.01
**Emergency admissions for any ACSC**													
Homeless (matched)	62,910	0.06 (0.65)	0	71	96.40	2.47	0.67	0.35	0.08	0.02	<0.01	<0.01	<0.01
Housed (matched)	292,842	0.03 (0.27)	0	19	97.56	1.91	0.33	0.16	0.03	0.01	0	0	0
Housed (before matching)	21,381,452	0.04 (0.29)	0	44	97.26	2.14	0.39	0.19	0.03	0.01	<0.01	<0.01	0
**Emergency admissions for acute ACSC**													
Homeless (matched)	62,910	0.03 (0.25)	0	10	97.52	1.91	0.40	0.15	0.02	<0.01	0	0	0
Housed (matched)	292,842	0.02 (0.16)	0	9	98.49	1.32	0.14	0.04	<0.01	0	0	0	0
Housed (before matching)	21,381,452	0.02 (0.17)	0	24	98.35	1.42	0.17	0.05	<0.01	<0.01	<0.01	0	0
**Emergency admissions for chronic ACSC**													
Homeless (matched)	62,910	0.03 (0.54)	0	61	98.54	0.95	0.27	0.17	0.04	0.01	<0.01	<0.01	<0.01
Housed (matched)	292,842	0.02 (0.21)	0	17	98.92	0.79	0.17	0.09	0.02	0.01	0	0	0
Housed (before matching)	21,381,452	0.02 (0.22)	0	44	98.73	0.95	0.19	0.10	0.02	<0.01	<0.01	<0.01	0

Data source: Hospital Episode Statistics Admitted Patient Care 2014/15-2017/18; ACSC = ambulatory care-sensitive condition

For peer review only - http://bmjopen.bmj.com/site/about/guidelines.xhtml

Table 3: Negative binomial regression estimates (Incident Rate Ratios) of the effect of homelessness on annual hospital admissions

	Admissions		Emergency admissions for an ACSC		
	Any (1)	Emergency (2)	Any ACSC (3)	Acute (4)	Chronic (5)
Homelessness	1.79***	2.08***	1.65***	1.78***	1.45***
	[1.69; 1.90]	[1.95; 2.21]	[1.51; 1.80]	[1.64; 1.93]	[1.27; 1.66]
Year of admission					
2014 (reference category)					
2015	0.82***	0.82***	0.87***	0.87**	0.86**
	[0.79; 0.84]	[0.79; 0.85]	[0.81; 0.93]	[0.80; 0.95]	[0.77; 0.96]
2016	0.71***	0.72***	0.87***	0.88**	0.82***
	[0.69; 0.74]	[0.69; 0.75]	[0.80; 0.93]	[0.81; 0.97]	[0.74; 0.92]
2017	0.68***	0.70***	0.84***	0.82***	0.84**
	[0.65; 0.71]	[0.67; 0.73]	[0.77; 0.91]	[0.75; 0.91]	[0.74; 0.95]
Sex					
Female	1.32***	1.11**	1.14*	1.21***	1.00
	[1.24; 1.40]	[1.04; 1.19]	[1.03; 1.26]	[1.10; 1.34]	[0.85; 1.19]
Age at time of attendance					
16 to 25 (reference category)					
26 to 35	1.19***	1.24***	1.40***	1.60***	1.11
	[1.09; 1.29]	[1.13; 1.36]	[1.21; 1.63]	[1.39; 1.84]	[0.84; 1.46]
36 to 45	1.57***	1.71***	1.98***	2.18***	1.74***
	[1.43; 1.72]	[1.55; 1.88]	[1.71; 2.28]	[1.90; 2.49]	[1.37; 2.22]
46 to 55	1.87***	1.99***	2.48***	2.18***	3.06***
	[1.70; 2.06]	[1.80; 2.21]	[2.14; 2.89]	[1.89; 2.53]	[2.40; 3.91]

For peer review only - http://bmjopen.bmj.com/site/about/guidelines.xhtml

56 to 65	1.99***	2.07***	3.31***	2.31***	5.07***
	[1.79; 2.21]	[1.84; 2.32]	[2.81; 3.91]	[1.96; 2.72]	[3.90; 6.60]
66 to 75	3.17***	3.49***	7.30***	4.73***	11.34***
	[2.42; 4.15]	[2.64; 4.63]	[4.69; 11.37]	[3.74; 5.99]	[6.33; 20.34]
n	355,752	355,752	355,752	355,752	355,752

*p<0.05; **p<0.01; ***p<0.001; Data source: Hospital Episode Statistics Admitted Patient Care 2014/15-2017/18; ACSC = ambulatory care-sensitive condition

For peer review only

For peer review only - http://bmjopen.bmj.com/site/about/guidelines.xhtml

Table 4: Count and percent of ambulatory care-sensitive emergency admissions for specific conditions

	Housed (% of total)	Homeless (% of total)
**Acute**		
Influenza, pneumonia, other vaccine preventable	829 (8%)	259 (6%)
Angina	108 (1%)	57 (1%)
Dehydration and gastroenteritis	833 (8%)	193 (5%)
Pyelonephritis and kidney/urinary tract infections	958 (10%)	180 (5%)
Perforated/bleeding ulcer	290 (3%)	82 (2%)
Cellulitis	1,148 (12%)	722 (18%)
Ear, nose and throat (ENT)	331 (4%)	50 (1%)
Dental	188 (2%)	71 (2%)
Convulsions and epilepsy	492 (5%)	497 (12%)
**Chronic**		
Chronic viral hepatitis B	2 (<1%)	0 (0%)
Diabetes	773 (8%)	322 (8%)
Anaemia	114 (1%)	38 (1%)
Epilepsy/dementia/atrial	1,410 (14%)	587 (14%)
Heart failure	302 (3%)	41 (1%)
Angina	378 (4%)	335 (8%)
Hypertension	57 (1%)	8 (<1%)
Bronchitis	1,109 (11%)	402 (10%)
Asthma	643 (6%)	156 (4%)
Total count of ACS emergency admissions	9,965	4,000
Number of patients	74,780	16,161

ACS = Ambulatory care-sensitive; Data source: Hospital Episode Statistics Admitted Patient Care 2014/15-2017/18; percentages are rounded to the nearest whole number and may not add to 100

For peer review only - http://bmjopen.bmj.com/site/about/guidelines.xhtml

Figure 1: The effect of homelessness on outcomes with 95% confidence intervals. Note: ACSC=ambulatory care-sensitive condition; Data source: HES APC 2014/15-2017/18; full regression results in Table 3

For peer review only

For peer review only - http://bmjopen.bmj.com/site/about/guidelines.xhtml

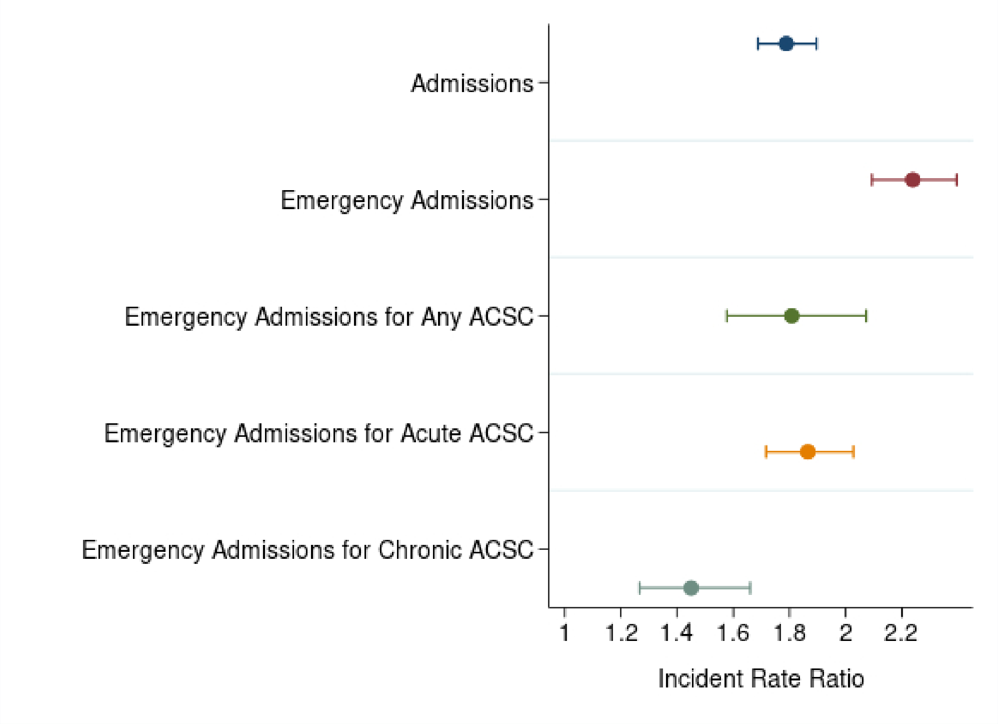

Figure 1: The effect of homelessness on outcomes with 95% confidence intervals. Note: ACSC=ambulatory care-sensitive condition; Data source: HES APC 2014/15-2017/18; full regression results in Table 3

205x149mm (72 x 72 DPI)

Supplementary Material

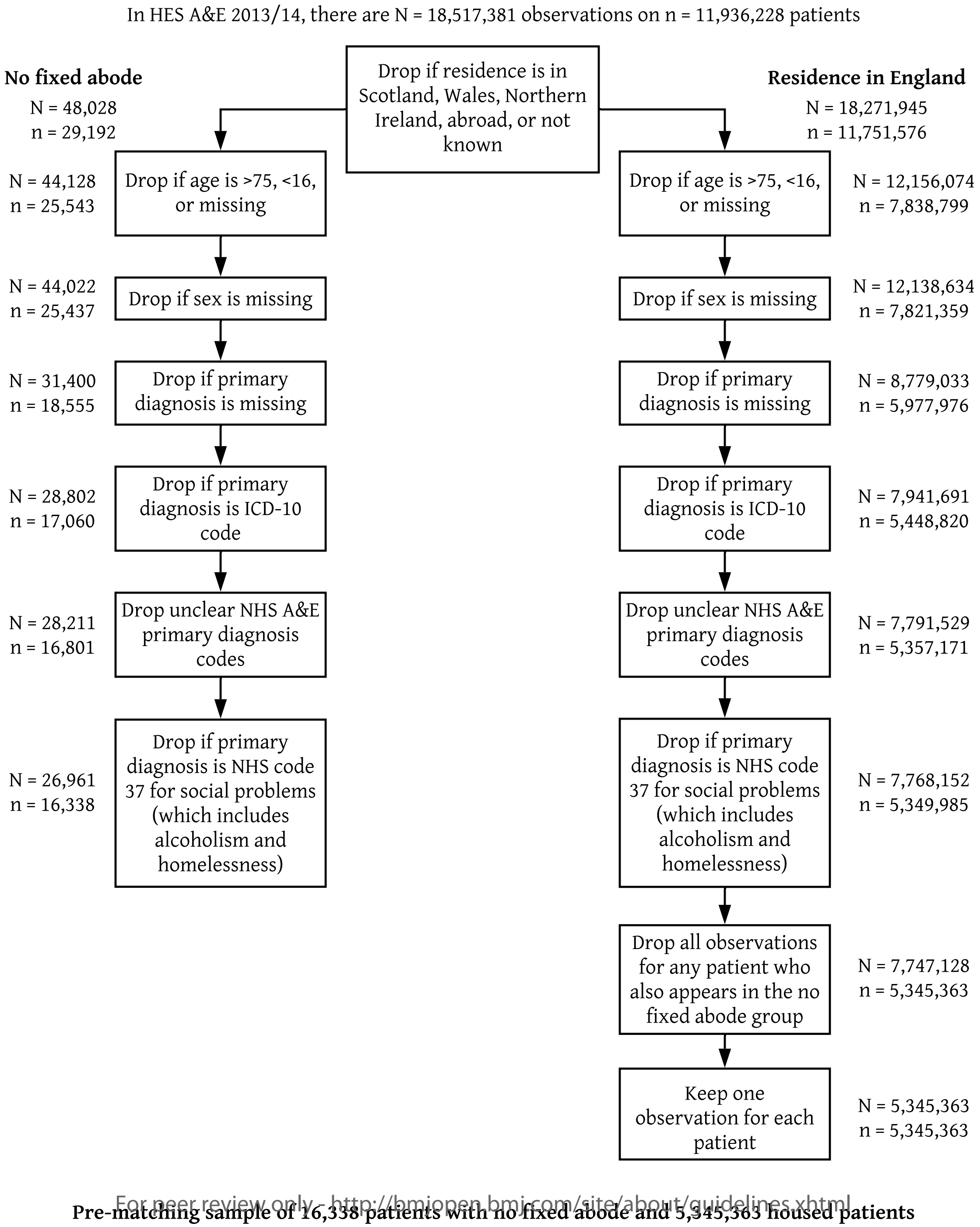

For peer review only - http://bmjopen.bmj.com/site/about/guidelines.xhtml

STROBE Statement—Checklist of items that should be included in reports of ***cohort studies***

Please note page numbers refer to version of manuscript without tracked changes

	Item No	Recommendation	How we have included this
**Title and abstract**	1	(*a*) Indicate the study's design with a commonly used term in the title or the abstract	We use the term longitudinal in the title (p.1) and explain our matching approach in the abstract (p.3)
		(*b*) Provide in the abstract an informative and balanced summary of what was done and what was found	Done (p.3)
**Introduction**			
Background/rationale	2	Explain the scientific background and rationale for the investigation being reported	We have referenced studies of homeless persons' health and access to health care (p.5)
Objectives	3	State specific objectives, including any prespecified hypotheses	We state our objectives in the final paragraph of the introduction (p.6)
**Methods**			
Study design	4	Present key elements of study design early in the paper	We do this in the abstract (p.3) and last paragraph of introduction (p.6)
Setting	5	Describe the setting, locations, and relevant dates, including periods of recruitment, exposure, follow-up, and data collection	Done, see methods section (p.6-9)
Participants	6	(*a*) Give the eligibility criteria, and the sources and methods of selection of participants. Describe methods of follow-up	Done, see methods section (p.6-9)
		(*b*) For matched studies, give matching criteria and number of exposed and unexposed	See methods section p.6-8. We have also included a flow chart as supplementary material.
Variables	7	Clearly define all outcomes, exposures, predictors, potential confounders, and effect modifiers. Give diagnostic criteria, if applicable	We have done this in the methods section (p.6-9)
Data sources/ measurement	8*	For each variable of interest, give sources of data and details of methods of assessment (measurement). Describe comparability of assessment methods if there is more than one group	We have explained and referenced our two sources of data in the methods section: HES on p.6 and ONS deaths data on p.8
Bias	9	Describe any efforts to address potential sources of bias	We have described our use of deaths data (p.8)
Study size	10	Explain how the study size was arrived at	Done in flowchart and methods section (p.6-8)
Quantitative variables	11	Explain how quantitative variables were handled in the analyses. If applicable, describe which groupings	Done in methods section (p.8-9)

For peer review only - http://bmjopen.bmj.com/site/about/guidelines.xhtml

		were chosen and why	
Statistical methods	12	(*a*) Describe all statistical methods, including those used to control for confounding	We described our matching and regression analysis (p.7-9)
		(*b*) Describe any methods used to examine subgroups and interactions	N/A - We didn't look at any subgroups within the two cohorts.
		(*c*) Explain how missing data were addressed	Done in methods section (p.6-7) and flowchart.
		(*d*) If applicable, explain how loss to follow-up was addressed	N/A
		(*e*) Describe any sensitivity analyses	N/A
**Results**			
Participants	13*	(a) Report numbers of individuals at each stage of study—eg numbers potentially eligible, examined for eligibility, confirmed eligible, included in the study, completing follow-up, and analysed	Done in flowchart in supplementary material.
		(b) Give reasons for non-participation at each stage	Number of observations dropped at each stage is in flowchart, reason for dropping is explained in methods (p.6-8)
		(c) Consider use of a flow diagram	Included as supplementary material
Descriptive data	14*	(a) Give characteristics of study participants (eg demographic, clinical, social) and information on exposures and potential confounders	We include demographic characteristics in Table 1 (p.19-20), descriptive statistics for outcomes in Table 2 (p.21).
		(b) Indicate number of participants with missing data for each variable of interest	We do not have any missing data for our outcomes. Number of observations dropped at each stage of sample selection are shown in flowchart in supplementary material
		(c) Summarise follow-up time (eg, average and total amount)	Done, it is the same for every patient (4 financial years)
Outcome data	15*	Report numbers of outcome events or summary measures over time	Done, Table 2 (p.21)
Main results	16	(*a*) Give unadjusted estimates and, if applicable, confounder-adjusted estimates and their precision (eg, 95% confidence interval). Make clear which confounders were adjusted for and why they were included	For each outcome, we include regression results with covariates (Table 3; p.22-23). We include confidence intervals in the results table and in the abstract (p.3) and main text (p.10)

For peer review only - http://bmjopen.bmj.com/site/about/guidelines.xhtml

		(*b*) Report category boundaries when continuous variables were categorized	We reported the age brackets that we used in the regressions (p.9 and results table on p.22-23)
		(*c*) If relevant, consider translating estimates of relative risk into absolute risk for a meaningful time period	N/A
Other analyses	17	Report other analyses done—eg analyses of subgroups and interactions, and sensitivity analyses	N/A
**Discussion**			
Key results	18	Summarise key results with reference to study objectives	Done in first section of discussion (p.10-11)
Limitations	19	Discuss limitations of the study, taking into account sources of potential bias or imprecision. Discuss both direction and magnitude of any potential bias	Done in limitations section (p.12-13)
Interpretation	20	Give a cautious overall interpretation of results considering objectives, limitations, multiplicity of analyses, results from similar studies, and other relevant evidence	Done in explanation section (p.11)
Generalisability	21	Discuss the generalisability (external validity) of the study results	Done in limitations section (p.12)
**Other information**			
Funding	22	Give the source of funding and the role of the funders for the present study and, if applicable, for the original study on which the present article is based	We have provided the sources of funding on p.2

*Give information separately for exposed and unexposed groups.

Note: An Explanation and Elaboration article discusses each checklist item and gives methodological background and published examples of transparent reporting. The STROBE checklist is best used in conjunction with this article (freely available on the Web sites of PLoS Medicine at http://www.plosmedicine.org/, Annals of Internal Medicine at http://www.annals.org/, and Epidemiology at http://www.epidem.com/). Information on the STROBE Initiative is available at http://www.strobe-statement.org.

For peer review only
